# Elevated lymphocyte specific protein 1 expression is involved in the regulation of leukocyte migration and immunosuppressive microenvironment in glioblastoma

**DOI:** 10.18632/aging.102706

**Published:** 2020-01-29

**Authors:** Jing-Yuan Cao, Qing Guo, Ge-Fei Guan, Chen Zhu, Cun-Yi Zou, Lu-Yang Zhang, Wen Cheng, Guo-li Wang, Peng Cheng, An-Hua Wu, Guang-Yu Li

**Affiliations:** 1 Department of Neurosurgery, The First Hospital of China Medical University, Shenyang, Liaoning 110001, China; 2 Department of Biochemistry, School of Life Science, China Medical University, Shenyang, Liaoning 110122, China; 3 College of Applied Technology, China Medical University, Shenyang, Liaoning 110122, China

**Keywords:** LSP1, glioblastoma, microenvironment, migration, immunosuppression

## Abstract

Immune cell infiltration mediates therapeutic response to immune therapies. The investigation on the genes regulating leukocyte migration may help us to understand the mechanisms regulating immune cell infiltration in tumor microenvironment. Here, we collected the data from Chinese Glioma Genome Atlas (CGGA) and The Cancer Genome Atlas (TCGA) to analyze the expression of leukocyte migration related genes in glioblastoma (GBM). Lymphocyte specific protein 1 (LSP1) was identified as the only gene in this family which not only has an elevated expression, but also serve as an independent predictive factor for progressive malignancy in glioma. We further confirmed these results in clinical glioma samples by quantitative PCR, immunofluorescence, immunohistochemistry, and western blot. Moreover, *LSP1* expression was closely related to the response to radio- and chemotherapy in GBM, and positively correlated with immunosuppressive cell populations, including M2 macrophages, neutrophil, and regulatory T cell. Additionally, elevated LSP-1 expression enhanced the expression of immunosuppression related genes like programmed cell death 1 (PD1) and leukocyte associated immunoglobulin like receptor 1 (LAIR1) in macrophages. LSP1 also promoted the migration of macrophages. Together, our study suggests a novel role of LSP1 contributing to immunosuppressive microenvironment in GBM and serving as a potential therapeutic target for it.

## INTRODUCTION

In recent years, researchers have obtained remarkable progress in the field of cancer immunotherapy, which provides new options for the treatment of cancers [1–3]. Dramatic responses have been observed across various tumor types with immunotherapy, particularly immune checkpoint inhibitors and chimeric antigen receptor (CAR) T cells [4, 5]. However, not all tumors are susceptible to current immunotherapy strategies, and even among those patients who do have a response, the effects are not durable [6, 7]. Thus, there is a critical unmet need to identify the mechanisms of response and resistance to immunotherapy, and design rational combination strategies [8, 9]. The understanding of immune response in tumor microenvironment need to be further improved, because of its complex and dynamic nature [10, 11].

Glioblastomas (GBM) is the most common primary malignant tumor in adult central nervous system and carries an abysmal 10.1% 3-year survival rate with standard care of surgery, radiation therapy and temozolomide chemotherapy [12]. New therapies are desperately needed for these patients. The immunosuppressive and cold phenotype of tumor microenvironment (TME) has been identified as a key regulator in GBM progression and recurrence [13]. TME in GBM is a unique challenge to treat, because tumor cell extrinsic components are native to the brain, as well as tumor intrinsic mechanisms which aid in immune evasion [14]. Targeting the genetically stromal components and reducing the immunosuppression caused by these cells is expected to convert the “cold” TME to a more “hot” TME phenotype, and may create new opportunities for GBM patients and circumvents the complications of tumor antigen directed therapies [14]. Recently, a clinical trial found that neoadjuvant anti-PD-1 immunotherapy promotes a survival benefit in recurrent glioblastoma [15]. The recruitment and function of different types of immune cells in the TME markedly change during tumor evolution in a manner that appears to be strongly context dependent [16, 17]. For example, tumor associated macrophages (TAMs) and neutrophils have been shown to produce pro-inflamatory cytokines, regulate glioma stem cell pools, and induce resistance to anti-angiogenic therapies [18, 19]. Understanding the mechanism of this process will help us find new TME-target strategies against GBM.

As a component of the podosome cap, Leukocyte-specific protein 1 (LSP1) is a myosin-associated regulator of macrophage phagocytosis and immune cell migration in inflammation [20, 21]. Aberrant LSP1 overexpression leads to reduced motility of neutrophils in the patients with neutrophil actin dysfunction [22]. LSP1 deficiency leads to enhanced T cell migration, and contributes to the development of rheumatoid arthritis [21]. In hematopoietic cells, LSP1 is an F-actin-binding protein that has a scaffold for the Ras-mitogen-activated protein kinase pathway and promotes leukocyte migration [23]. Loss of LSP1 expression leads to enhanced skin wound healing, suggesting a role for LSP1 in cell proliferation [24, 25]. However, the expression pattern of LSP1 and its role in cancer biology and the regulation of the TME remain to be further explained.

Therefore, in present study, we first summarized a list of leukocyte migration related genes. Then we investigated the expression pattern of these genes in GBM and identified *LSP1* as the leukocyte migration related gene with the most correlated with GBM patient. Second, we explored the value of *LSP1* as a prognostic molecule in glioma with data from Chinese Glioma Genome Atlas (CGGA) and The Cancer Genome Atlas (TCGA). The expression of LSP1 was further verified with quantitative PCR (qPCR), immunohistochemistry and western blot in clinical tissue samples. In addition, we verified the potential of LSP1 as an independent risk factor for glioma malignancy and a therapeutic molecule for targeted strategies of glioma. Moreover, function annotation of *LSP1* in GBM showed its function in strengthening the local immune response and mediating immune suppression in GBM. The analyses on the correlation between *LSP1* and immune cell populations in GBM’s TME revealed that *LSP1* was significantly positive correlation with M2 macrophages, T regulatory (Treg) and neutrophils, and negatively correlated with cytotoxic lymphocytes. LSP1 also showed a close expressive relevance with immune checkpoint genes like PD-1 and promoted the migration of macrophages. Taken together, this study suggests LSP1 as a contributor of immunosuppressive TME in GBM and a possible therapeutic target in developing new therapeutic immune strategies in GBM.

## RESULTS

### The analysis of leukocyte migration related genes in glioma identifies *LSP1* as an independent risk factor for progressive malignancy in glioma

**Figure 1:**
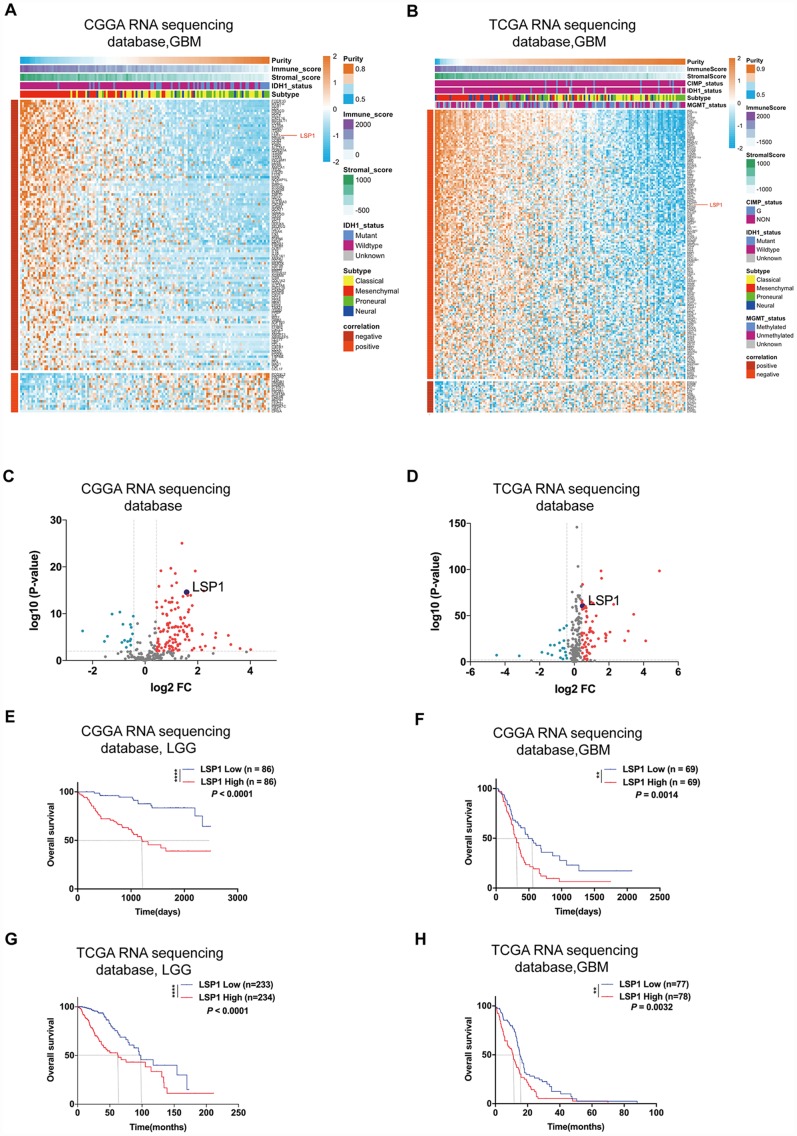
***LSP1* was the only gene in leukocyte migration related genes, which not only had an elevated expression, but also was correlated with unfavorable prognosis in glioma.** (**A** and **B**) Heatmaps describing the correlation between the expression of leukocyte migration related genes and tumor purity, immune and stroma scores, and *IDH*1 status in GBM (**A**, CGGA RNA sequencing dataset, n = 138; **B**, TCGA RNA sequencing dataset, n = 155). (**C** and **D**) Volcano plots showing differentially expressed leukocyte migration related genes between GBM and LGG (**C**, CGGA RNAseq, n = 310; **D**, TCGA RNAseq, n = 622; with *t* test). (**E**–**H**) Kaplan-Meier survival analyses revealed an association between high *LSP1* expression and unfavorable outcomes in both of LGG and GBM (**E**, **F**: CGGA RNA sequencing dataset; **G**, **H**: TCGA RNA sequencing dataset; with log-rank test). **, and **** indicate no significance *P* < 0.01, and *P* < 0.0001, respectively. CGGA, Chinese Glioma Genome Atlas; TCGA, The Cancer Genome Atlas; GBM, glioblastoma multiforme; LSP1, lymphocyte specific protein 1; LGG, lower-grade glioma.

**Figure 2:**
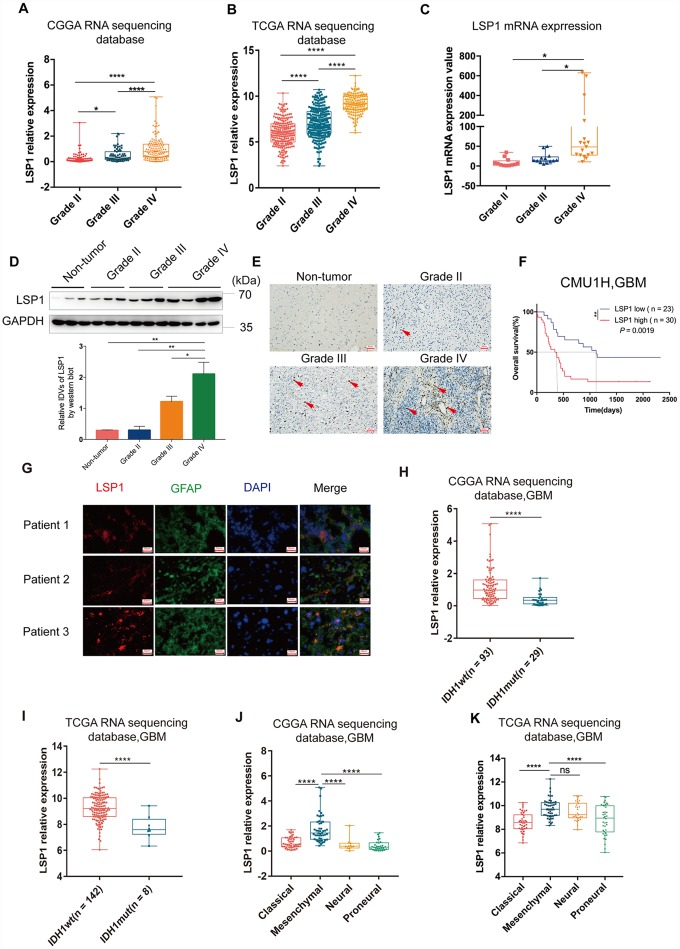
**The analyses of *LSP1* expression according to WHO grades, GBM subtypes and *IDH1* mutant status.** (**A** and **B**) *LSP1* expression was significantly increased in GBM in comparison with WHO grade II and WHO grade III glioma (**A**, CGGA RNA sequencing dataset, Grade II n = 105; Grade III n = 67; Grade IV n = 138; **B**, TCGA RNA sequencing dataset, Grade II n = 224; Grade III n = 243; Grade IV n = 155; with one-way ANOVA). (**C**) qPCR analysis of *LSP1* expression in clinical glioma samples (Grade II n = 11; Grade III n = 10; Grade IV n = 21; with one-way ANOVA). (**D**) Representative western blot images (upper panel) and analyses of LSP1 (lower panel) expressed in clinical tissues (Non-tumor n = 3; Grade II n = 3; Grade III n = 3; Grade IV n = 3; with one-way ANOVA). (**E**) Representative IHC images of LSP1 staining in clinical non-tumor and glioma samples (200X, scale bar = 50μm). (**F**) Kaplan-Meier curve evaluating the association of LSP1 expression with the prognosis of GBM patients (LSP1 high vs. low, *P* = 0.0019; with log-rank test). (**G**) Representative IF co-staining images of LSP1 and GFAP in clinical GBM samples (200X, scale bar = 50μm). (**H** and **I**) *LSP1* expression was significantly upregulated in *IDH1* wild type GBM (**H**, CGGA RNA sequencing dataset; **I** TCGA RNA sequencing dataset; with *t* test). (**J** and **K**) The expression analysis of *LSP1* in four subtypes of GBM (**J**, CGGA RNAseq, Classical n = 47, Mesenchymal n = 50, Neural n = 11, Proneural n = 30; **K**, TCGA RNAseq, Classical n = 40, Mesenchymal n = 50, Neural n = 27, Proneural n = 38; with one-way ANOVA). ns, *, ** and **** indicate no significance, *P* < 0.05, *P* < 0.01, and *P* < 0.0001, respectively. CGGA, Chinese Glioma Genome Atlas; TCGA, The Cancer Genome Atlas; GBM, glioblastoma multiforme; LSP1, lymphocyte specific protein 1; GFAP, glial fibrillary acidic protein; DAPI, 4’,6-diamidino-2-phenylindole; IDH1, isocitrate dehydrogenase 1.

GBM’s microenvironment has been suggested to be a major determinant responsible for tumor recurrence and high lethality of GBM patients. The “cold” TME of GBM is characterized with relatively few tumor infiltrating lymphocytes (TILs) [26]. As leukocyte migration plays a key role in the distribution of immune cells throughout the body [27], the investigation on the expression of leukocyte migration related genes in GBM may help us identify the gene responsible for the regulation of immune cell infiltration in GBM. Based on these observations, we first summarized a list of leukocyte migration related genes (Supplementary Table 1) [23, 28] and analyzed the correlation between these genes and clinical pathological features, including tumor purity, immune score, stromal score, isocitrate dehydrogenase 1(*IDH1*) status, and subtypes, with CGGA and TCGA GBM RNA sequencing datasets. We found that most of genes related to leukocyte migration were significantly associated with glioma purity, including 1 overlapping positively purity-related gene and 21 overlapping negatively purity-related genes (Figure 1A, 1B; *P* < 0.05, r > 0.4 or r < -0.6) (Supplementary Table 2). Furthermore, we compared the expression pattern of these genes in low grade glioma (LGG) and GBM (*P* < 0.05, log_2_FC > 0.37) with CGGA and TCGA RNA sequencing data. The result showed that there were 61 overlapping differentially expressed genes in both datasets (Figure 1C, 1D and Supplementary Table 3). Combined these data, there were 8 leukocyte migration related genes (Supplementary Table 4), which were not only highly associated with glioma purity, but also differentially expressed between LGG and GBM. To compare the prognostic relevance of these 8 genes, we further performed a univariate Cox regression analysis with the survival data from CGGA and TCGA. The result demonstrated that *LSP1* was the only gene significantly correlated with a poor prognosis in GBM (*P* < 0.01, Supplementary Table 4). We further examined the prognostic value of *LSP1* expression in LGG and GBM with log-rank test. The data also demonstrated that patients with higher *LSP1* expression had a significantly shorter survival times than their counterparts in both of LGG and GBM (Figure 1E–1H and Supplementary Figure 1A–1D). Therefore, we chose *LSP1* as a further research target. Due to prominent heterogeneity of molecular nature across different grades of glioma, *LSP1* expression was analyzed according to the 2016 WHO grade system. According to CGGA and TCGA, GBM showed the highest *LSP1* expression in comparison to grade II and grade III glioma (Figure 2A, 2B, and Supplementary Figure 2A). Furthermore, we verified this result in clinical glioma samples with qPCR, western blot and IHC, and similar result was obtained (Figure 2C–2E). Additionally, we investigated the LSP1 expression level in benign tissue around LSP1 high tumor by IHC. The result showed that benign tissue around LSP1 high tumor had a significant lower LSP1 expression level than tumor tissue (Supplementary Figure 2B). The data of immunofluorescence colocalization showed that there were a few cells in GBM samples with co-staining of LSP1 and glial fibrillary acidic protein (GFAP), which may imply a tumor cell-related LSP1 expression in GBM (Figure 2G). Finally, we confirmed that higher LSP1 expression related to a shorter survival in GBM with clinical samples from our hospital with IHC (Figure 2F and Supplementary Table 5). Taken together, these data indicate the potential of LSP1 as an independent predicative factor for progressive malignancy in glioma and high LSP1 expression predicts unfavorable survival in glioma.

### High *LSP1* expression was enriched in *IDH1* wild type and mesenchymal subtype of GBM

Next, investigation was performed with *IDH* mutation status as a sub-classifier. Mutation in IDH1 is a stable marker for better prognosis in both lower-grade glioma (LGG) and glioblastoma multiforme (GBM) [29]. As the earliest detectable genetic alteration in gliomagenesis, IDH1 heterozygous missense mutations in codon 132 cause an arginine-to-histidine substitution in 80–90 % of cases (R132H) [30] that leads to a distinct metabolism and hypermethylation phenotype in gliomas [31]. IDH1 mutations caused down-regulation of leukocyte chemotaxis, resulting in repression of the tumor-associated immune system [32]. Given that significant infiltration of immune cells such as macrophages, microglia, monocytes, lymphocyte, and neutrophils is linked to poor prognosis in many cancer types, these disrupted immune infiltrates in IDH1 mutation glioma tumors may contribute to the different aggressiveness of these two glioma types. Therefore, we compared LSP1 expression between IDH1 mutation and wild type. The result indicated that GBM with wild type *IDH1* presented a higher level of *LSP1* expression (Figure 2H, 2I and Supplementary Figure 2C–2E). This suggested that elevated *LSP1* expression was more common in *IDH1* wild-type glioma and further reflected different biological genetic background between these two kinds of tumors. Moreover, we found that mesenchymal GBM exhibited a higher expression level of *LSP1* than another three subtypes (proneural, classical, and neural) (Figure 2J, 2K and Supplementary Figure 2F–2H). We further employed ROC curve and AUC measurement to examine the sensitivity and specificity of LSP1 as a marker for mesenchymal GBM. The result confirmed the potential of *LSP1* to distinguish mesenchymal subtype in GBM (Supplementary Figure 2I, 2J). Altogether, these findings indicated that LSP1 expression was elevated in GBM, especially in *IDH1* wild type and mesenchymal subtype tumors.

### *LSP1* predicts radiotherapeutic and chemotherapeutic response in GBM patients

**Figure 3:**
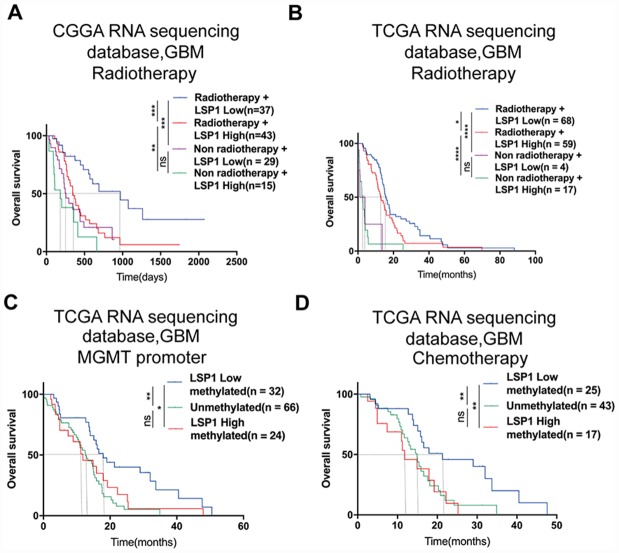
***LSP1* exhibited the potential as a molecule for predicting radiotherapeutic and chemotherapeutic response in GBM.** (**A** and **B**) Kaplan-Meier curves describing the association between *LSP1* expression and the survival of GBM patients treated with or without radiotherapy (**A**, CGGA RNA sequencing dataset; **B**, TCGA RNA sequencing dataset; with log-rank test). (**C**) Kaplan-Meier curve describing the correlation between *LSP1* expression and the survival of GBM patients with different *MGMT* promoter status in TCGA RNA sequencing dataset (with log-rank test). (**D**) Kaplan-Meier curve describing the correlation between *LSP1* expression and the survival of GBM patients receiving chemotherapy in TCGA RNA sequencing dataset (with log-rank test). ns, *, **, *** and **** indicate no significance, *P* < 0.05, *P* < 0.01, *P* < 0.001, and *P* < 0.0001, respectively. CGGA, Chinese Glioma Genome Atlas; TCGA, The Cancer Genome Atlas; GBM, glioblastoma multiforme; LSP1, lymphocyte specific protein 1; MGMT, O^6^-methylguanine-DNA methyltransferase.

Previous studies indicated that LSP1 expression was associated with malignant biologic process in malignancies like breast cancer and Hodgkin’s disease [33, 34]. Since these malignant biological behaviors have been reported to contribute to radiotherapy and chemotherapy resistance [35], we employed multivariate Cox regression and survival analyses to examine whether LSP1 could serve as a marker for the prediction of the response to radiotherapy and chemotherapy in GBM patients. The result of multivariate Cox regression analysis revealed that *LSP1* expression was significantly associated with the survival of GBM patients with radio- and chemo-therapy (Supplementary Table 6). Moreover, according to treatment strategies (whether to receive radiation therapy) and LSP1 expression, we divided the samples in CGGA and TCGA into four groups, including high LSP1 expression with or without radiotherapy and low LSP1 expression with or without radiotherapy. We found that no matter whether *LSP1* expression in GBM patients was high or low, patients receiving radiotherapy had a longer survival times compared to those without radiotherapy (Figure 3A, 3B and Supplementary Figure 3A, 3B). But the low LSP1 group had a survival advantage compared to high group in GBM patients receiving radiotherapy, but not those without radiotherapy (Figure 3A, 3B and Supplementary Figure 3A, 3B). This suggested that LSP1 might participant in mediating radiotherapy resistance in GBM patients. O^6^-methylguanine-DNA methyltransferase (MGMT) promoter methylation has been identified as a predictive marker for GBM patients treated with temozolomide (TMZ) chemotherapy, and Higher level of *MGMT* promoter unmethylation lead to TMZ resistance [36]. The analyses of *LSP1* expression in three TCGA GBM datasets did not show consistent results between *MGMT* promoter methylated and unmethylated group. *LSP1* had a higher expression in *MGMT* promoter unmethylated group in two microarray datasets, but not in RNA sequencing dataset (Supplementary Figure 3C–3E). We further analyzed the association between *LSP1* expression and the survival of GBM patients with different *MGMT* promoter status. Based on *MGMT* promoter status and *LSP1* expression, the samples in TCGA were divided into three groups, including *MGMT* promoter methylated with high or low *LSP1* expression and *MGMT* promoter unmethylated. The result showed that the low *LSP1* group had a survival advantage compared to high group with methylated *MGMT* promoter (Figure 3C and Supplementary Figure 3F, 3G). In contrary, there was no survival difference between *MGMT* promoter unmethylated and methylated group with higher expression of *LSP1*, which suggested higher expression of *LSP1* could eliminate the prognosis advantage of *MGMT* promoter methylation (Figure 3C and Supplementary Figure 3F, 3G). Lastly, the analyses with the data from three TCGA datasets showed that high *LSP1* expression with methylated *MGMT* promoter had no significant survival advantage over unmethylated group in chemotherapy patients (Figure 3D and Supplementary Figure 3H, 3I). This suggested that *LSP1* mediating chemotherapy resistance in GBM may be closely related to *MGMT* promoter methylation. Collectively, these data indicate that *LSP1* could serve as a molecule for the response prediction to radiotherapy and chemotherapy in GBM.

### Functional enrichment analyses reveal that *LSP1* is associated with immunologic events

**Figure 4:**
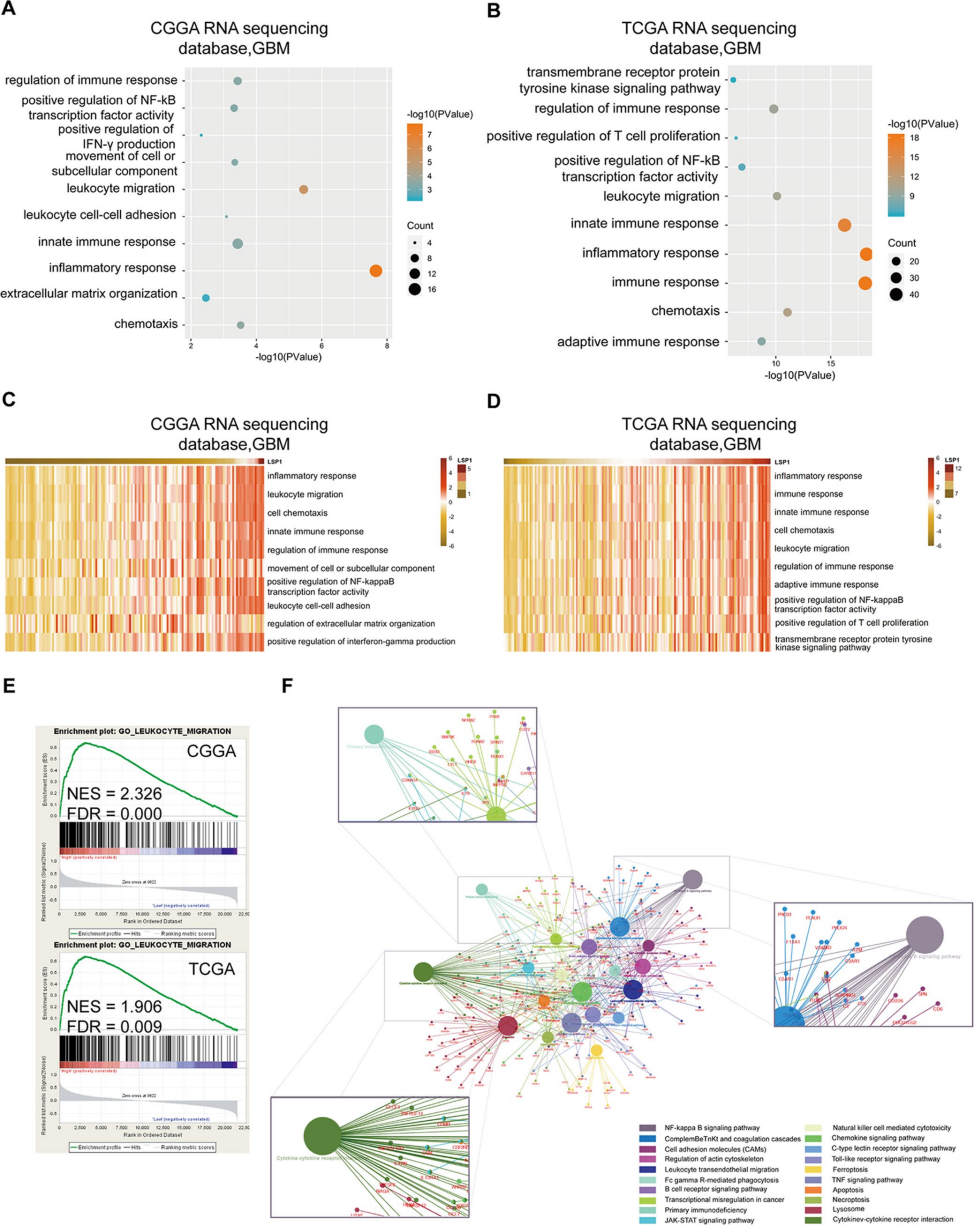
**Functional enrichment analysis revealed that *LSP1* was associated with immunologic events.** (**A** and **B**) The results of GO analysis describing biological processes associated with *LSP1* positive-correlated genes in GBM (**A**, CGGA RNA sequencing dataset; **B**, TCGA RNA sequencing dataset). Bubble diameter: enrichment gene counts; abscissa: -log 10 *P*-value (*P* < 0.05). (**C** and **D**) The GO terms correlated with high *LSP1* expression in GBM by GSVA (**C**, CGGA RNA sequencing dataset; **D**, TCGA RNA sequencing dataset). (**E**) The results of GSEA indicating a significantly enhanced leukocyte migration in GBM with high *LSP1* expression (upper panel, CGGA RNA sequencing dataset; lower panel, TCGA RNA sequencing dataset; *P* < 0.05 and FDR < 0.01). (**F**) The *LSP1* related pathways revealed by 892 overlapping *LSP1* positively related genes in CGGA and TCGA RNA sequencing datasets with ClueGO. CGGA, Chinese Glioma Genome Atlas; TCGA, The Cancer Genome Atlas; GBM, glioblastoma multiforme; LSP1, lymphocyte specific protein 1.

Next, we focus on exploring the functional role of *LSP1* in GBM. A list of genes positively correlated with *LSP1* expression (Pearson r > 0.5, and *P* < 0.05) was obtained from CGGA and TCGA GBM RNA sequencing datasets, respectively (Supplementary Table 7). GO analysis was performed based on this gene list. And the result showed that genes most relevant to *LSP1* were mostly involved in the regulation of leukocyte migration, immune response, and inflammatory response (Figure 4A, 4B). While genes that negatively correlated with *LSP1* expression (r < -0.4, and *P* < 0.05) contributed to the regulation of normal biological process, such as brain and spinal cord development (Supplementary Figure 4A, 4B). Additionally, in consistent with the above data, the results of GSVA showed the enrichment of leukocyte migration, inflammatory response, and the regulation of immune response phenotypes in samples with high *LSP1* expression (Figure 4C, 4D). GSEA also demonstrated a close association between *LSP1* expression and the regulation of leukocyte migration, inflammatory response, and immune response in GBM (Figure 4E and Supplementary Figure 4C, 4D). Moreover, we summarized the overlapping up-regulated genes correlated with high *LSP1* expression in CGGA and TCGA RNA sequencing datasets (Pearson r > 0.3, and *P* < 0.05). There were 892 overlapping up-regulated genes (Supplementary Table 8). As shown in Figure 4F, the result of KEGG pathway analysis showed that LSP1 was significantly correlated to immune related pathways, such as leukocyte trans-endothelial migration, NF-kappa B signaling pathway, cytokine-cytokine receptor interaction, and primary immunodeficiency. These findings imply that LSP1 may play an important role in regulating immunologic biological processes of GBM.

### High *LSP1* expression is accompanied by increasing macrophage, neutrophil and Treg cell infiltrating GBM

**Figure 5:**
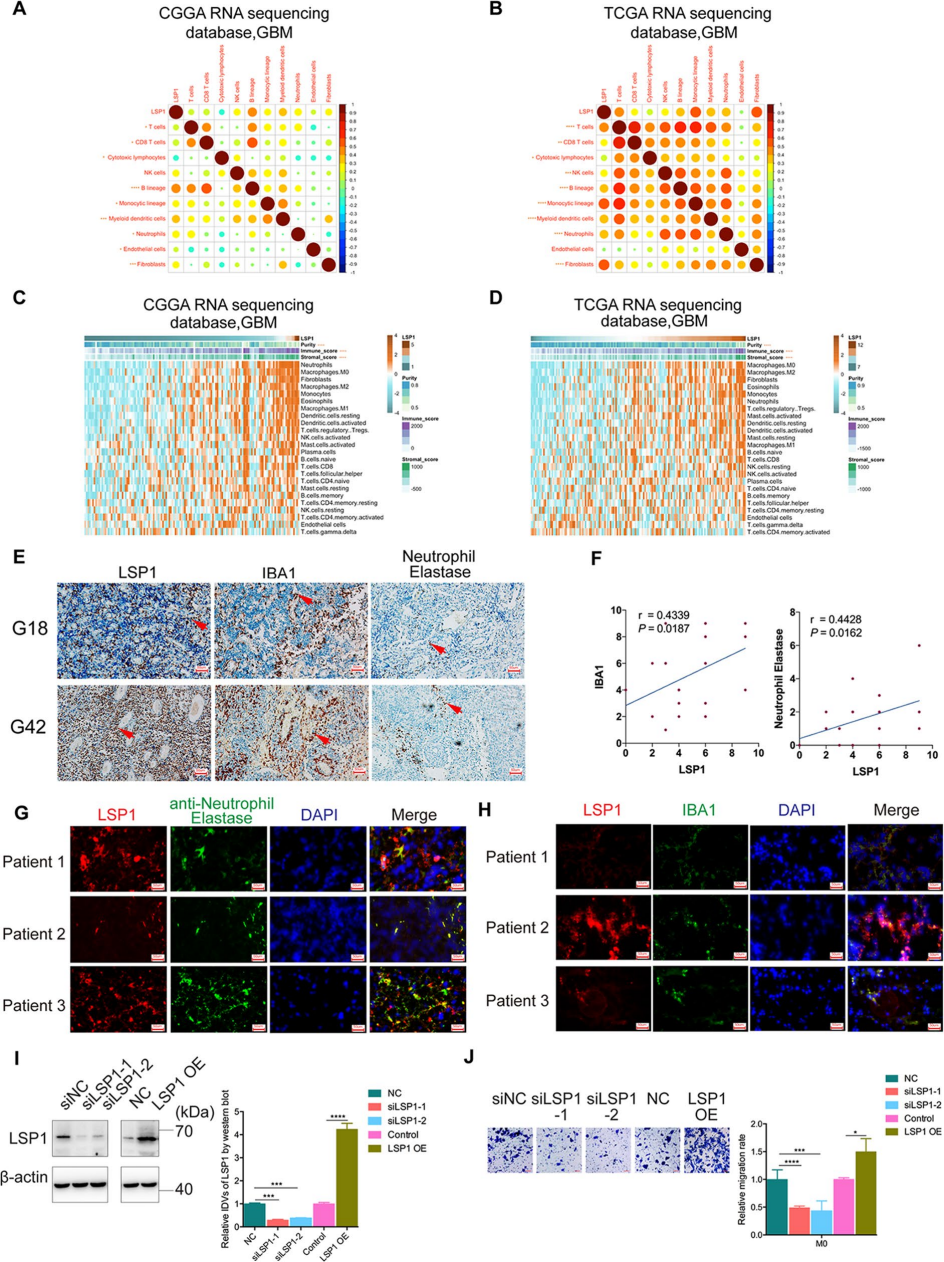
**High *LSP1* expression contributed to the immunosuppressive microenvironment in GBM.** (**A** and **B**) The correlation analysis of *LSP1* expression and non-tumor immune and stromal cell populations in GBM by MCP-counter (**A**, CGGA RNA sequencing dataset, n = 138; **B**, TCGA RNA sequencing dataset, n = 155; with Pearson correlation analysis). (**C** and **D**) Association of *LSP1* expression with tumor purity, immune and stromal score, and twenty-four immune cell populations in GBM microenvironment by GSVA. (**C**, CGGA RNA sequencing dataset, n = 138; **D**, TCGA RNA sequencing dataset, n = 155; with Pearson correlation analysis). (**E** and **F**) Representative IHC images (**E**, 200X, scale bar = 50μm) and analysis (**F**) verifying LSP1 expression correlated with macrophages and neutrophil in 29 cases of GBM samples (macrophage: r = 0.4339, *P* = 0.0187; neutrophil: r = 0.4428, *P* = 0.0162; n = 15; with Pearson correlation analysis). (**G**) Representative IF images of LSP1 (red), Neutrophil Elastase (green), and DAPI (blue) staining in clinical GBM samples (n = 3) (200X, scale bar = 50μm). (**H**) Representative IF images of LSP1 (red), IBA1 (green), and DAPI (blue) staining in clinical GBM samples (n = 3) (200X, scale bar = 50μm). (**I**) Representative western blot image (left panel) and analysis (right panel) of LSP1 expression in M0 macrophages induced from THP-1 cells. (**J**) Transwell assay showing LSP1 knockdown inhibit the migration of M0 macrophages, and LSP1 overexpression enhanced their migration. CGGA, Chinese Glioma Genome Atlas; TCGA, The Cancer Genome Atlas; GBM, glioblastoma multiforme; LSP1, lymphocyte specific protein 1; IBA1, ionized calcium binding adapter molecule 1; DAPI, 4’,6-diamidino-2-phenylindole; IDH1, isocitrate dehydrogenase 1.

The TME of glioma contains noncancerous cell types including immune and stroma cells, which may promote tumor progression and mediate therapeutic resistance via extensive crosstalk with glioma cells in the TME [13]. Through mutual relationship analysis of *LSP1* expression and noncancerous cells with MCP-counter method, we found that *LSP1* expression was strongly negatively associated with cytotoxic lymphocytes, and positively correlated with B lineage, monocytic lineage neutrophils, and fibroblasts in GBM TME (Figure 5A, 5B). To further explore the relation between *LSP1* and different nontumor cell populations in the TME, we analyzed the enrichment scores of 24 noncancerous cell types by GSVA (Figure 5C, 5D) [37]. The result demonstrated that *LSP1* was negatively related with cytotoxic lymphocytes and positively related with Tregs, neutrophils and macrophage, especially M2 macrophage. Tregs are generated from the bone marrow and the thymus. In glioma patients, there is an increased proportion of immunosuppressive Tregs within the remaining CD4 + cell pool in blood, and a prominent infiltrating Treg population within GBM tumor tissue [13]. Neutrophils come from the bone marrow and are mobilized into the blood during inflammation. Neutrophils in glioma tissue are infiltrated from blood.

Additionally, TAMs in GBM are monocyte-derived macrophages from peripheral blood. Macrophages can be categorized into M1 and M2 subtypes based on their polarization status. Glioma cell could recruit M2 tumor-associated macrophages and promote their growth [38]. Furthermore, our previous research revealed that macrophage and neutrophil indicated poor prognosis in glioma patient [39]. Thus, we further performed IHC staining of LSP1, IBA1 (macrophage marker) and Neutrophil Elastase (neutrophil marker) in 29 clinical GBM samples. There were73.33% (11/15) high macrophage and 66.67% (10/15) high neutrophil infiltrated in LSP1-high GBM tissue samples. Simultaneously, 71.43% (10/14) low macrophage infiltration and 64.29% (9/14) low neutrophil infiltration in LSP1-low GBM tissue (Figure 5E, 5F). Moreover, the result of immunofluorescence colocalization confirmed that LSP1 was expressed in neutrophils and macrophages in GBM tissue (Figure 5G, 5H). This matched the above results of GO analysis, GSVA and GSEA. We further investigated the level of LSP-1 expression in glioma cells (U87, LN229, T98, and PGC21) and non-tumor cells (NHA, THP1(M0), THP1 induced M1 and M2 cells, and PBMC). The results showed that the expression levels of LSP1 in THP1(M0), M1 and M2 macrophages induced from THP1 cells, and PBMC were significantly higher than that in tumor cells (Supplementary Figure 5A, 5B). Thus, we proposed that LSP1 mainly functioned with non-tumor cell population in GBM. Based on this observation, we investigated the effect of LSP1 in M0 macrophages induced from THP1 cells on GBM migration abilities. As shown in Figure 5I and 5J, LSP1overexpression in M0 macrophages induced from THP1 cells increased their migration abilities. But LSP1 overexpression in U87 and PGC21 (a primary adherent glioma cell line from a clinical GBM sample) glioma cells didn’t increased their migration abilities (Supplementary Figure 5C, 5D). Together, these results indicate that LSP1 might contribute to the immunosuppressive response in GBM, and regulate the behaviors of immune cells like macrophages (M2).

### The up-regulated expression of immunosuppressive genes and LSP1 is a major feature in GBM

**Figure 6:**
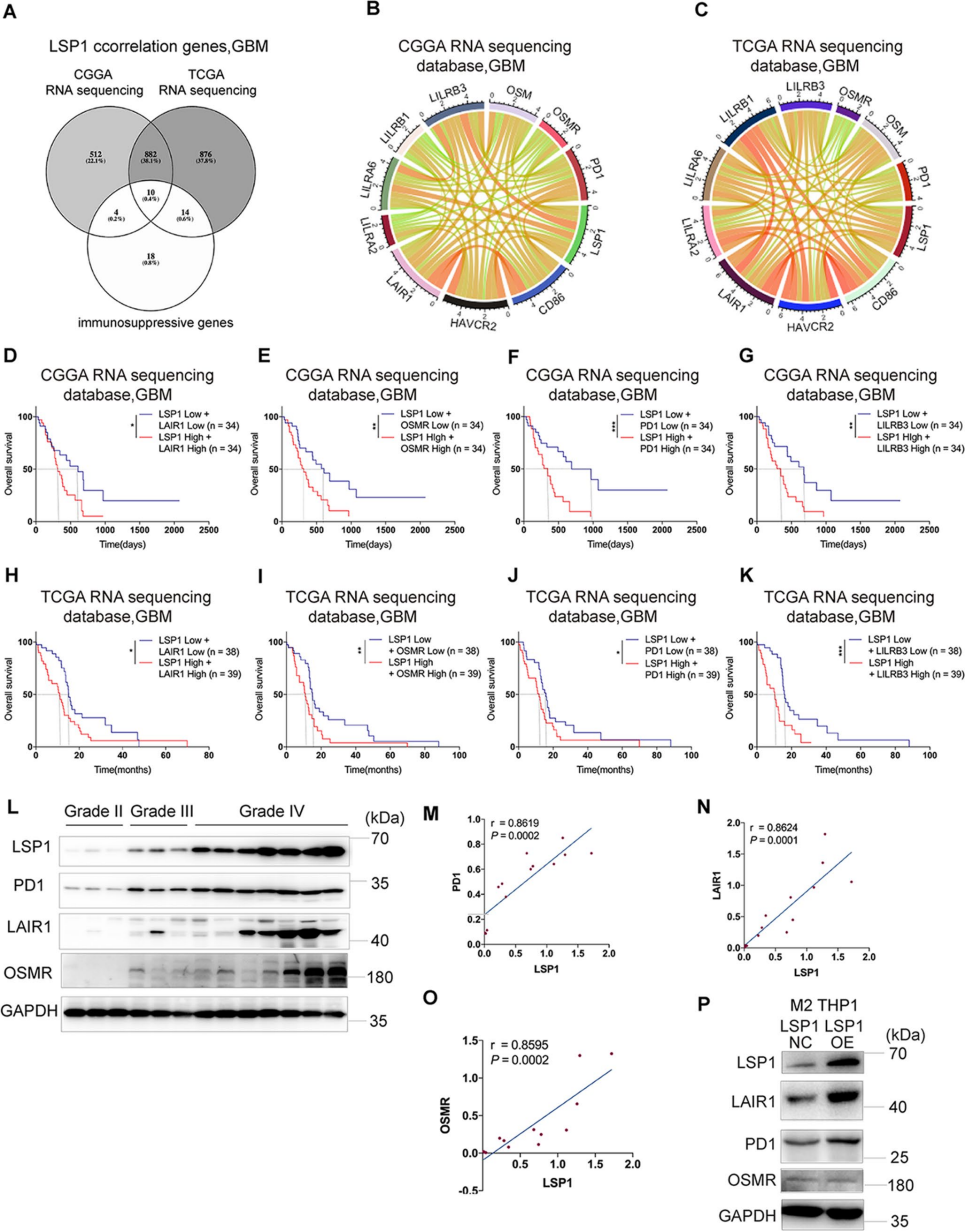
**The expression of immunosuppressive genes is a major feature in GBM with high LSP1 expression.** (**A**) A venn diagram showing the 10 overlapping immunosuppressive genes positively correlated with high *LSP1* expression in CGGA and TCGA RNA sequencing datasets, and immunosuppressive gene sets. (*P* < 0.05; with *t* test). (**B** and **C**) Correlation analyses of *LSP1* and the above 10 immunosuppressive genes in GBM (**B**, CGGA RNA sequencing dataset, n = 138; **C**, TCGA RNA sequencing dataset, n = 155; with Pearson correlation analysis). (**D**–**K**) Kaplan-Meier curves describing combined prognostic value of *LSP1* and *LAIR1*, *OSMR*, *PD1*, *LILRB3* expression in GBM (**D**–**G**, CGGA RNA sequencing dataset; **H**–**K**, TCGA RNA sequencing dataset; with log-rank test). (**L**) Representative western blot images of LSP1, PD1, LAIR1 and OSMR in clinical tissues (Grade II n = 3; Grade III n = 3; Grade IV n = 7). (**M**–**O**) The correlation analysis of LSP1 with PD1 (**M**), LAIR1 (**N**) or OSMR (**O**) in clinical tissues with western blot (PD1: r = 0.8619, *P* = 0.0002; LAIR1: r = 0.8624, *P* = 0.0001; OSMR: r = 0.8595, *P* = 0.0002; with Pearson correlation analysis). (**P**) Representative western blot images showing LSP1 overexpression significantly increased PD1 and LAIR1 expression in M2 macrophages induced from THP-1 cells. *, ***, and **** indicate *P* < 0.05, *P* < 0.001, and *P* < 0.0001, respectively. *, **, and *** indicate *P* < 0.05, *P* < 0.01, and *P* < 0.001, respectively. CGGA, Chinese Glioma Genome Atlas; TCGA, The Cancer Genome Atlas; GBM, glioblastoma multiforme.

Last, on the basis of above results, we investigated the correlation between *LSP1* expression and immunosuppressive genes in GBM (Supplementary Table 9). The result demonstrated strong correlations between *LSP1* and the following molecules, including *OSM*, *OSMR*, *PD1*, *CD86*, *HAVCR2*, *LAIR1*, *LILRA2*, *LILRA6*, *LILRB1* and *LILRB3* (Figure 6A–6C). This matched with the analysis of *LSP1*-immune-related pathways (cytokine-cytokine receptor interaction) in the CCGA and TCGA RNA sequencing datasets (Figure 4F). Furthermore, we evaluated the prognosis value of the combination of *LSP1* and *OSM*, *OSMR*, *PD1*, *CD86*, *HAVCR2*, *LAIR1*, *LILRA2*, *LILRA6*, *LILRB1*, and *LILRB3* expression. We found that *LAIR1*, *OSMR*, *PD1*, and *LILRB3* had the prognosis value of the combination with *LSP1* in the CGGA and TCGA datasets in GBM, and the co-upregulation of *LSP1* and these genes is a predictor of poor survival in GBM patients, respectively (Figure 6D–6K). Due to the important roles of PD1, LARI1, and OSMR contributing to immunosuppressive microenvironment, we further examined the co-expression of LSP-1 and PD1, LARI1, and OSMR in clinical different grades glioma samples by IHC (Figure 6L). We found a correlation between LSP-1 expression and these genes (Figure 6O). In addition, we performed LSP1 overexpression in M2 macrophages induced from THP-1 cells. We found that elevated LSP1 expression increased their LAIR1 and PD1 expression, but not OSMR (Figure 6P). Collectively, these data further support a crucial role of LSP1 in the regulation of immune response in GBM TME.

## DISCUSSION

Immune evasion is a hallmark of carcinogenesis [40]. Gaining insight into the biology of immunosuppressive TME in GBM may disclose new therapeutic target for this devastating disease. In this study, among the genes related to leukocyte migration, we found that LSP1 was the only gene which not only had an elevated expression, but also was associated with poor survival in patients with GBM or LGG. We confirmed the potential of *LSP1* as a progressive malignancy marker in glioma. *LSP1* expression also had a close association with *IDH1* wild type tumor, and could be used as an indicator for the survival of GBM patient with radio- and chemotherapy. Finally, *LSP1* was associated with immunologic events in GBM. Elevated LSP1 expression promoted macrophage migration and enhanced the expression of immunosuppressive molecules like PD1 and LAIR1 in macrophages.

It is well known that the wild type and mutant form of *IDH1* have important impact on the regulation of local immune response and tumorigenesis in glioma [32]. The mutant form of *IDH1* attenuated leukocyte chemotaxis, resulting in the repression of local immune system and leaded to immune suppression in GBM TME [32]. Our results revealed that LSP1 had a low expression in *IDH1* mutant GBM, which reached the same conclusion with the above report. Future study is needed to further investigate the association and related mechanism between *IDH1* and *LSP1*. Radio- and chemotherapy were the two main treatment strategies for GBM patients after tumor resection, but not all patients could benefit from radiation or chemotherapy. Through our analysis, low *LSP1* expression indicated the sensitivity of radio- or chemotherapy in GBM. This may help us to improve individual treatment strategies for GBM patients.

Immune suppression has been recognized as a main characteristic in glioma. Previous study reported that cancer cells could evade destruction through upregulation of immune-checkpoint ligands, such as PD-L1, which can bind complementary receptors on immune cell and cause suppression of lymphocyte activation [7]. We found that the high level of *LSP1* expression was closely associated with multiple immune response signaling pathways (Figure 4). LSP1 expression was also significantly correlated with the expression of ten immune regulatory genes, *PD1*, *HAVCR2*, *LILRA2*, *LILRB1*, *LILRB3*, *LILRB6*, *LAIR1*, *CD86, OSMR*, and *OSM*. Because of the important roles of these molecules in mediating immunosuppression, the strong correlations between *LSP1* and these molecules may imply a potential role of *LSP1* in mediating local immune response. In addition, *LSP1* expression was positively associated with the immunosuppressive cell subpopulations, like neutrophils, Tregs, and M2 macrophages and negatively associated with cytotoxic lymphocytes. Since these immunosuppressive cells lead to cytotoxicity CD8+ T cells “exhaustion” [41], LSP1 expression in these cell subpopulations may contribute to the “cold tumor” status of GBM. Additionally, we observed the expression of PD1 and LAIR1 is upregulated in response to LSP1 overexpression in M2 macrophages from THP1 cells. The migration of M0 macrophages was also increased by LSP1 overexpression. These data further support LSP1 contribution to immunosuppression TME in GBM. Future study is needed to clarify the mechanism of their interactions.

In conclusion, according to clinical samples, and multiple dataset profiles, we first revealed an elevated LSP1 expression in GBM compared to LGG. Second, we confirmed the correlation between upregulated LSP1 expression and unfavorable patients’ survival, and increased *LSP1* expression was associated with the progressive malignancy in diffuse glioma. Third, we showed the potential of *LSP1* as a molecule to predict the response of GBM patients to radiotherapy and chemotherapy. Finally, we find a previous undefined role of *LSP1* in the regulation of local immune response in GBM which may contribute to the high lethality of GBM. This may disclose a new aspect to explain the “cold” status of GBM. Taken together, these findings imply the potential of *LSP1* as a candidate target in developing novel immune strategies against glioma. Our study may contribute to improving the understanding of the properties and functions of TME in GBM, and help to develop new treatment strategies against glioma.

## MATERIALS AND METHODS

### Human specimens and ethics

This study was approved by the Ethics Committee of the First Hospital of China Medical University. All the clinical samples used in this study were collected at the First Hospital of China Medical University from January, 2011 to March, 2019, including 42 samples (11 cases for grade II, 10 cases for grade III, and 21 cases for grade IV tissue samples) for qPCR, 16 samples (3 cases for non-tumor, 3 cases for grade II, 3 cases for grade III, and 7 cases for grade IV tissue samples) for western blot and 90 samples for immunohistochemistry (5 cases for non-tumor, 7 cases for grade II, 17 cases for grade III, and 61 cases for grade IV, respectively) in which 53 cases of grade IV (GBM) samples for IHC had survival information. The histological diagnoses of these samples were confirmed by two neuropathologists, according to the 2016 World Health Organization (WHO) classification guidelines. The samples were de-identified before processed to laboratories. Informed consent was obtained from each patient.

### Cell culture

U87 cells were purchased from GeneChem (Shanghai, China). Human normal astrocytes (NHA) and LN229 cells were obtained from Beijing Neurosurgical Institute. NHA, U87, and LN229 cells were maintained in Dulbecco’s modified Eagle’s medium (DMEM, Gibco) containing 10% fetal bovine serum (FBS, Gibco) and 1% penicillin/streptomycin (Gibco) at 37°C with 5% CO_2_. Patient-derived primary adherent glioma cells (PGC21) was derived from fresh glioma bulk immediately after operation in the First Hospital of China Medical University, which is cultured in RPMI-1640 medium (Gibco), containing 10% FBS and 1% penicillin/streptomycin (Gibco) at 37°C with 5% CO_2_. The identities of PGC21 has been authenticated by short tandem repeat (STR) analysis. Human peripheral blood mononuclear cell (PBMC) were isolated by Ficoll-Paque PLUS (GE Healthcare) centrifugation media from health donor’s blood as previously described [38]. THP1 cells were provided by Professor Xin Meng (Department of Biochemistry, China Medical University). THP1 monocytes were primed with 5nM PMA (Sigma) for 48 hours to become monocyte-derived macrophages [42]. M1 phenotype macrophages were activated with lipopolysaccharide (LPS), while M2 phenotype were polarized with interleukin 4 (IL4) [43].

### RNA isolation and reverse-transcription quantitative PCR (RT-qPCR)

TRIzol reagent (Invitrogen) was used for RNA isolation. Total RNA was reversely transcribed into cDNA with Prime-Script RT Master Mix (TaKaRa). qPCR was carried out in a thermal cycler (PCR LightCycler 480, Roche) with SYBR Green Master Mix (TaKaRa) as previously described [44]. The primer sequences were as follows: LSP1 (Forward primer: AGGACCGAGTCCCTAAACCG, Reverse primer: CTGGGTGTATTGTTCCAGCCA); GAPDH (Forward primer: GGAGCGAGATCC CTCCAAAAT, Reverse primer: GGCTGTTGTCATACTTCTCATGG). The mRNA expression of target genes was calculated by the 2^-ΔΔCT^ method and normalized to GAPDH mRNA expression [45].

### Protein extraction and western blotting

Total protein from each sample was obtained and separated as previously reported [46]. Then the protein was transferred to PVDF membranes (Millipore), followed by 1 hour 5% skimmed milk blocking at room temperature and incubated overnight at 4°C with primary antibodies (LSP1 1:1000, Santa Cruz, sc-53363; OSMR 1:1000, Proteintech, 10982-1-AP; LAIR1 1:1000, Santa Cruz, sc-398141; PD1 1:1000, Proteintech, 66220-1-lg; or GAPDH 1:1000, Proteintech, 10494-1-AP). Secondary antibody incubation was performed with peroxidase-conjugated affinipure goat anti-mouse IgG or anti-rabbit IgG (Proteintech; 1:5000). Protein bands were visualized with chemiluminescence ECL reagents (Tanon) and quantified using Image J software.

### Immunohistochemistry (IHC)

The IHC staining and the quantification of staining intensity was performed as previously described with the following primary antibodies respectively (LSP1, Santa Cruz, sc-53363; IBA1, abcam, ab5076; Neurophil Elastase, abcam, ab68672) [44, 46].

### Immunofluorescence (IF)

For immunocytochemistry, 4μm thick section slides were prepared from clinical samples. Then the sections were permeabilized with 0.5% Triton X-100 for 20 min. After 5% BSA incubation for 1 h, primary antibody (LSP-1, Santa Cruz, sc-53363; GFAP, Proteintech, 16825-1-AP; Neurophil Elastase, abcam, ab6867; IBA1, abcam, ab5076) was added and incubated at 4°C overnight. Following incubation with fluorescein (FITC) or rhodamine (TRITC) secondary antibody and 4’,6-diamidino-2-phenylindole (DAPI), the samples were detected using fluorescence microscope (Leica DMi8).

### Data collection for *LSP1* expression, survival, and function analysis

The following transcriptome datasets from patients diagnosed with glioma (WHO II-IV) were employed for *LSP1* expression, survival, and function analyses: CGGA datasets (RNAseq: n = 310; mRNA microarray: n = 298) (http://www.cgga.org.cn), and TCGA datasets (RNAseq: n = 625; 4502A mRNA microarray: n = 488; U133 mRNA microarray: n = 525) (http://gliovis.bioinfo.cnio.es/) [47]. Only the samples with expression and survival information were included.

### Gene ontology (GO) and kyoto encyclopedia of genes and genomes (KEGG) analysis

After Pearson correlation analysis, gene ontology (GO) analysis of the genes positively related with high expression of *LSP1* was constructed in two datasets of GBM, CGGA RNAseq and TCGA RNAseq, respectively. GO analysis were performed with DAVID 6.8 (https://david.ncifcrf.gov/tools.jsp) [48]. Then, the overlapping upregulated genes associated with high level of *LSP1* expression were summarized from CGGA and TCGA RNAseq datasets, GBM. The relevant signaling pathways of high level of *LSP1* expression from KEGG were analyzed by ClueGO [49].

### Gene set enrichment analysis (GSEA) and gene set variation analysis (GSVA)

GSEA (http://www.broadinstitute.org/gsea/index.jsp) was applied to investigate *LSP1* associated biological function. Normalized enrichment score (NES) and false discovery rate (FDR) were used to determine the statistical significances according to a previous report [50]. GSVA (http://www.bioconductor.org) was used to further validate the association between *LSP1* and the candidate functions. GSVA was also implemented to investigate the relationship between 24 types of non-tumor cell subpopulations and *LSP1* expression.

### Transwell assay

Transwell assay was performed with 8μm inserts (Corning, 3422) as previously described [51].

### Statistical analysis

R language (version 3.5.2; R packages, including pheatmap, corrplot, and circus) and GraphPad Prism 7 software (version 7.0) were used for statistical analyses and generating figures, unless mentioned elsewhere. Statistical significance was defined as *P* value < 0.05. Significant quantitative differences between and among groups were determined by two-tailed *t* test and one-way ANOVA, respectively. The univariate and multivariate Cox regression analyses were performed for evaluating the prognostic variables. A Kaplan-Meier survival analysis was used to estimate the survival distribution, using the median value as the cutoff. Genes that showed differential expression between GBM and LGG cohorts from the two datasets were extracted by volcano plot using GraphPad Prism 7. The ROC curve was plotted, and the area under the ROC curve (AUC) of each cutoff was measured by GraphPad Prism 7.

## Supplementary Material

Supplementary Figures

Supplementary Tables 1-6, 9

Supplementary Table 7

Supplementary Table 8
