# Supplementary material for: Elevated lymphocyte specific protein 1 expression is involved in the regulation of leukocyte migration and immunosuppressive microenvironment in glioblastoma
Source: Aging (Albany NY). 2020 Jan 29;12(2):1656–84. doi: 10.18632/aging.102706 (PMC7053627; doi:10.18632/aging.102706)
Supplement: Supplementary Figures [file aging-12-102706-s004..pdf]

SUPPLEMENTARY FIGURES

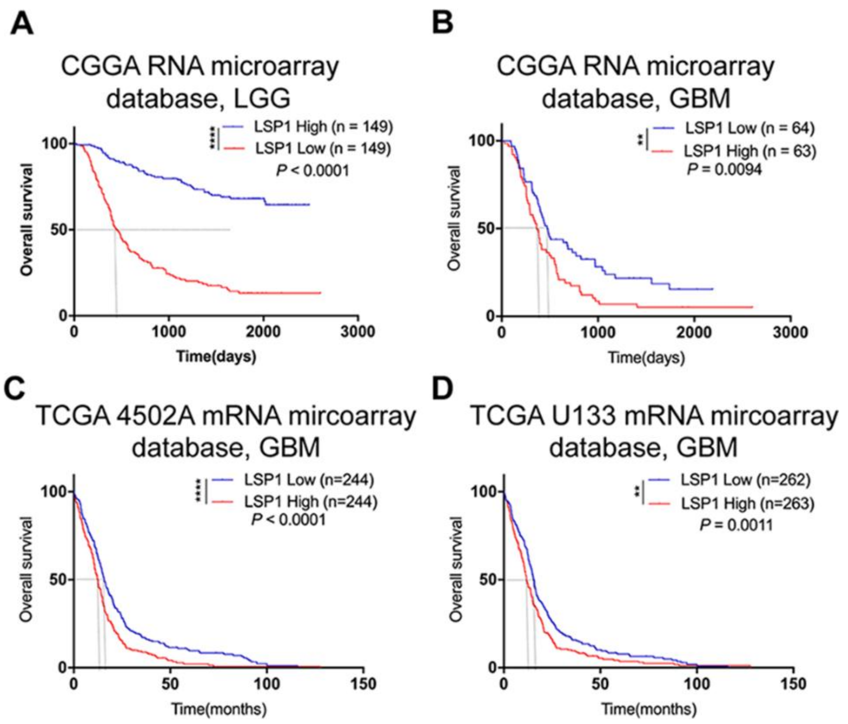

Supplementary Figure 1. High level of *LSP1* expression correlates with the unfavorable prognosis in glioma. (**A**–**D**) Kaplan-Meier plots revealed an association between high *LSP1* expression and unfavorable outcomes in LGG (**A**, CGGA mRNA microarray; with log-rank test) and GBM patients; **B**, CGGA mRNA microarray; **C**, TCGA-4502A mRNA microarray; **D**, TCGA-U133 mRNA microarray; with log-rank test). **, and **** indicate $P < 0.01$, and $P < 0.0001$, respectively. LGG, lower-grade glioma; GBM, glioblastoma multiforme.

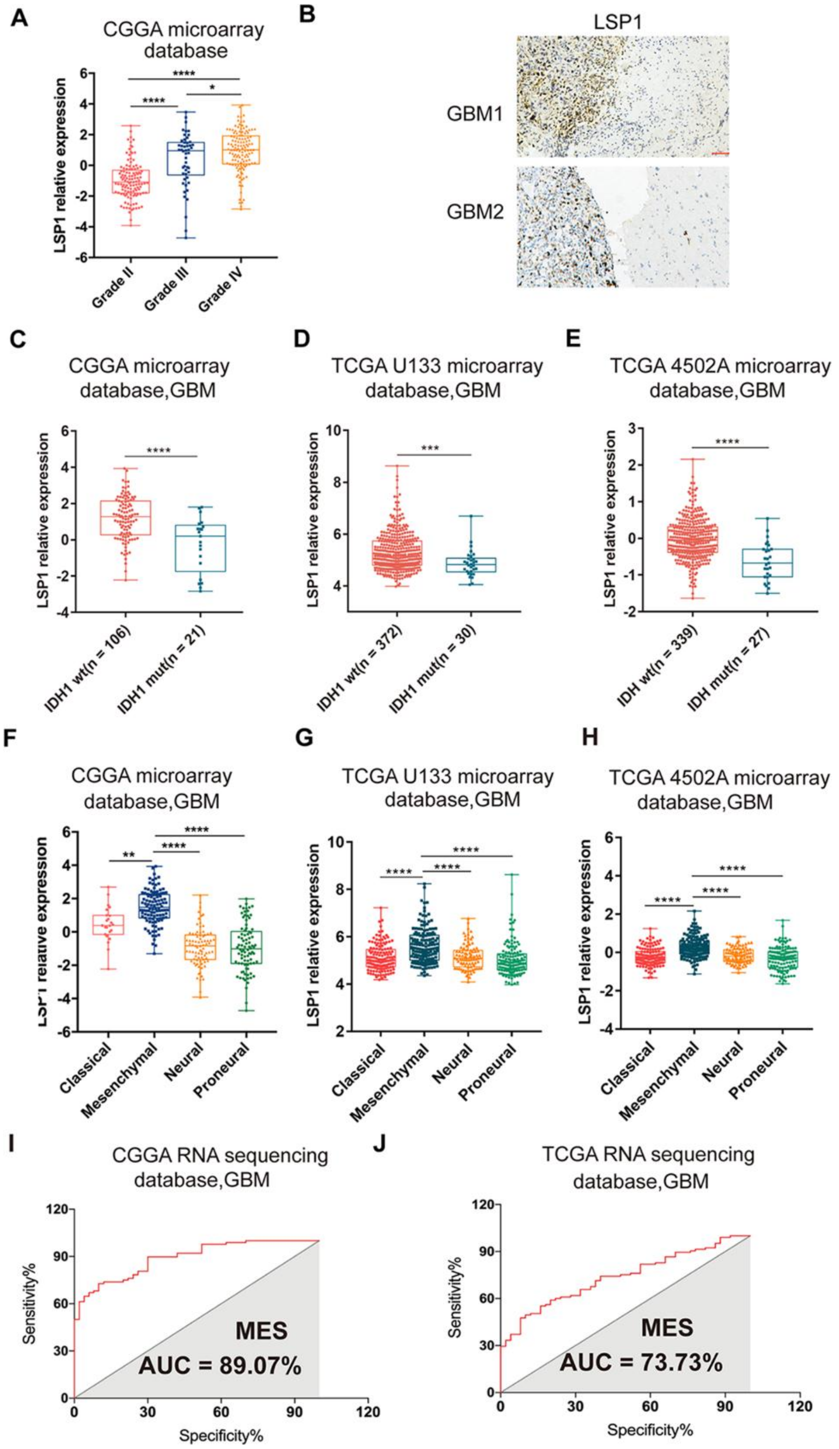

Supplementary Figure 2. *LSP1* expression was upregulated in GBM, especially mesenchymal subtype. (**A**) LSP1 expression was significantly higher in GBM than in grade II and III samples (Grade II n = 121, Grade III n = 50, Grade IV n = 127; with one-way ANOVA). (**B**) A representative image of LSP1 staining in GBM tissue including the benign tissue around tumor (200X, scale bar = 50μm). (**C**–**E**) *LSP1* was significantly upregulated in *IDH1* wild type gliomas of CGGA and TCGA datasets, GBM (**C**, CGGA mRNA microarray; **D**, TCGA-U133 mRNA microarray; **E**, TCGA-4502A mRNA microarray; with *t* test). (**F**–**H**) The expression pattern of *LSP1* in four subtypes of GBM (**F**, CGGA mRNA microarray, Classical n = 23, Mesenchymal n = 108, Neural n = 81, Proneural n = 86; **G**, TCGA-U133 mRNA microarray, Classical n = 144, Mesenchymal n = 156, Neural n = 88, Proneural n = 137; **H**, TCGA-4502A mRNA microarray, Classical n = 130, Mesenchymal n = 147, Neural n = 84, Proneural n = 127； with one-way ANOVA). (**I**, **J**) The sensitivity and specificity of LSP1 as a molecule for mesenchymal GBM (**I**, CGGA RNA sequencing dataset, n = 138; **J**, TCGA RNA sequencing dataset, n = 155; with ROC curve). *, **, *** and **** indicate $P < 0.05$, $P < 0.01$, $P < 0.001$, and $P < 0.0001$, respectively. GBM, glioblastoma multiforme; MES, mesenchymal subtype.

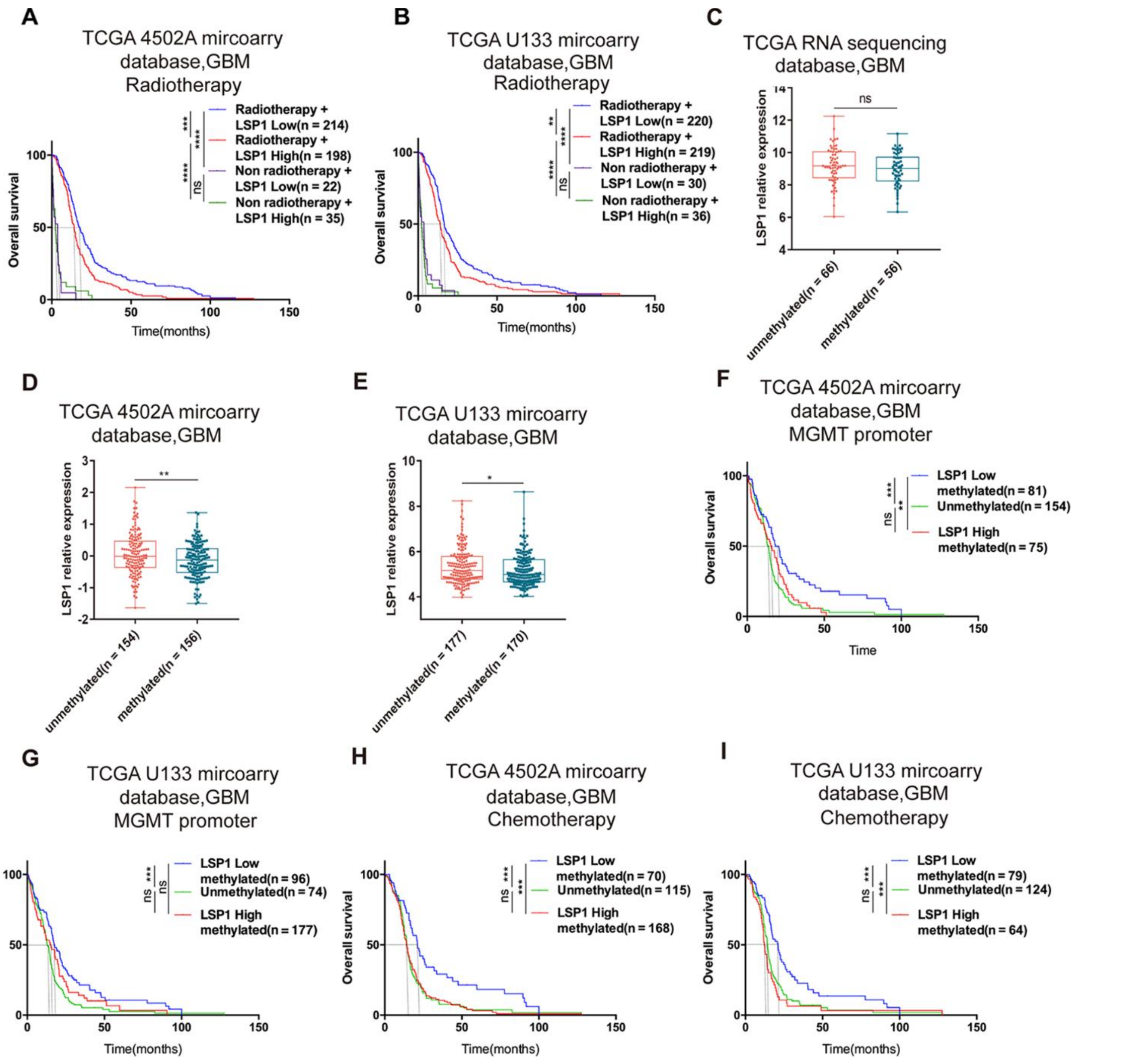

Supplementary Figure 3. The value of high *LSP1* expression in the response prediction to radio- and chemotherapy in GBM. (**A** and **B**) Kaplan-Meier curves described the association between *LSP1* expression and the survival of GBM patients treated with or without radiotherapy (**A**, TCGA-4502A mRNA microarray; **B**, TCGA-U133 mRNA microarray; with log-rank test). (**C**–**E**) The comparison of *LSP1* expression analyzed in GBM with or without *MGMT* promoter methylation patients (**C**, TCGA RNA sequencing dataset; **D**, TCGA 4502A microarray; **E**,TCGA U133 microarray; with *t* test). (**F** and **G**) Kaplan-Meier curves describing the correlation between *LSP1* expression and the survival of GBM patients with different *MGMT* promoter status (**F**, TCGA 4502A microarray; **G** TCGA U133 microarray; with log-rank test). (**H** and **I**) Kaplan-Meier curves described the correlation between *LSP1* expression and the survival of GBM patients receiving chemotherapy (**H**, TCGA 4502A microarray; **I**, TCGA U133 microarray; with log-rank test). ns, *, **, ***, and **** indicate no significance, $P < 0.05$, $P < 0.01$, $P < 0.001$, and $P < 0.0001$, respectively. GBM, glioblastoma multiforme; MGMT, O^6-methylguanine-DNA methyltransferase.

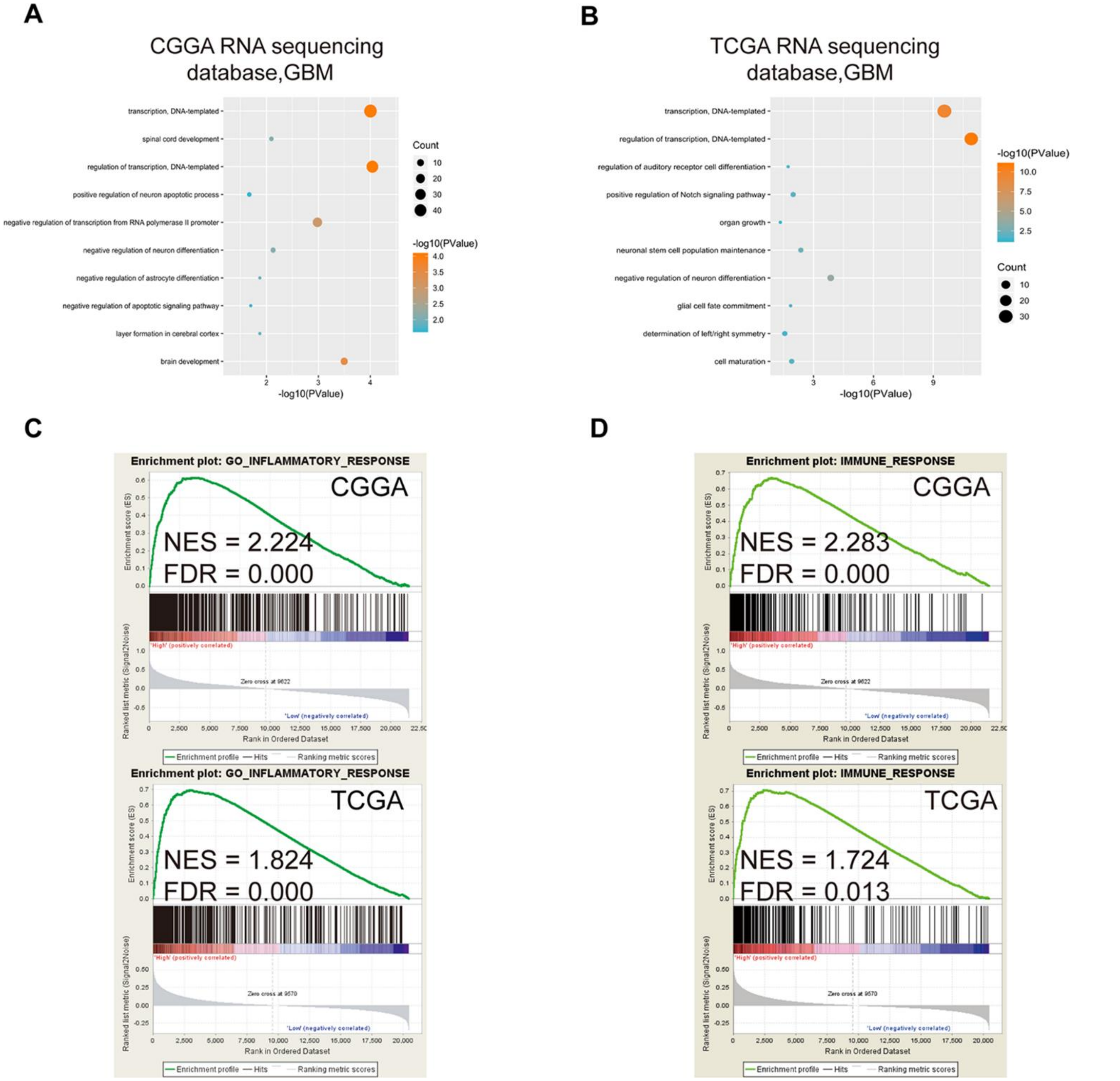

Supplementary Figure 4. Functional enrichment analysis revealing the association of LSP1 with immunologic events. (**A** and **B**) Bubble chart described the biological processes associated with *LSP1*-negative-correlated genes by GO analysis in GBM (**A**, CGGA RNA sequencing dataset; **B,** TCGA RNA sequencing dataset). Bubble diameter: enrichment gene counts; abscissa: -log 10 *P*-value $P < 0.05$. (**C** and **D**) GSEA indicated a significantly enhanced immune response and inflammatory response in GBM patients with high *LSP1* expression (**C**, CGGA RNA sequencing dataset; **D**, TCGA RNA sequencing dataset). $P < 0.05$ and FDR < 0.01. GBM, glioblastoma multiforme.

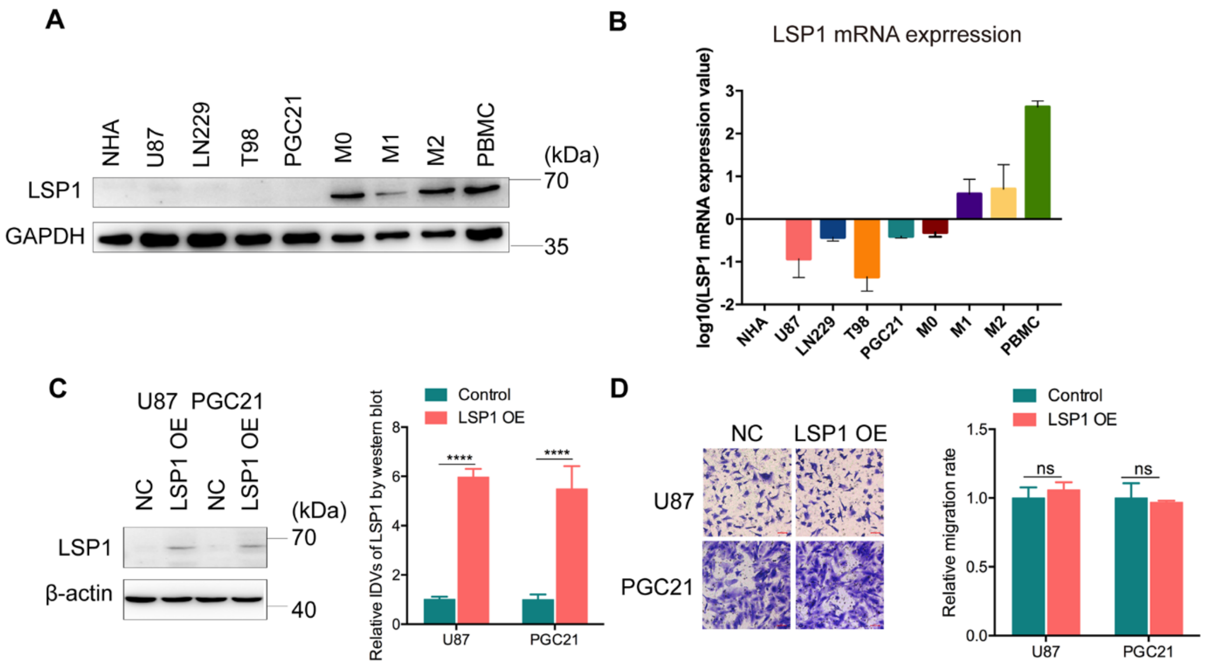

Supplementary Figure 5. Knockdown or overexpression of LSP1 didn't change the migration abilities of glioma cells. (**A**) Representative western blot image of LSP1 protein expression in indicated cell lines. (**B**) Quantitative RT-PCR analysis of LSP1 mRNA expression in indicated cell lines. (**C**) Representative western blot image (left panel) and analysis (right panel) of LSP1 expression in indicated glioma cells. (**D**) Transwell assay showing LSP1 knockdown or overexpression didn't affect the migration of indicated glioma cells. *, ***, and **** indicate *P* < 0.05, *P* < 0.001, and *P* < 0.0001, respectively.
