# Supplementary material for: Elevated lymphocyte specific protein 1 expression is involved in the regulation of leukocyte migration and immunosuppressive microenvironment in glioblastoma
Source: Aging (Albany NY). 2020 Jan 29;12(2):1656–84. doi: 10.18632/aging.102706 (PMC7053627; doi:10.18632/aging.102706)
Supplement: Supplementary Tables 1-6, 9 [file aging-12-102706-s003..pdf]

SUPPLEMENTARY TABLES

Supplementary Table 1. The list of leukocyte migration related genes.

ADAM8	CCR7	FCER1G	ITGB3	PPBP	THBD
AIMP1	CD177	FER	ITGB7	PPIA	TNF
AMICA1	CD2	FFAR2	JAGN1	PPIL2	TNFRSF11A
ANGPT1	CD244	FLT1	JAM2	PREX1	TNFSF11
ANGPT2	CD34	FN1	JAM3	PROC	TREM1
ANGPT4	CD44	FOLR2	KIT	PROCR	TRPM4
ANO6	CD47	FOXJ1	KRAS	PROS1	UMOD
ANXA1	CD48	FUT7	L1CAM	PTPN11	VAV1
APOB	CD58	FYN	LBP	PTPN6	VAV3
ARHGEF5	CD74	GAS6	LCK	PTPRO	VCAM1
ARTN	CD84	GATA3	LEP	RAC1	VEGFA
ATP1B1	CDC42	GBF1	LGALS3	RET	XCL1
ATP1B2	CEACAM1	GCNT1	LSP1	ROCK1	XCL2
ATP1B3	CEACAM6	GLG1	LYN	RPS19	YES1
AZU1	CEACAM8	GOLPH3	LYST	S100A12	ZAP70
B4GALT1	CHGA	GP6	MADCAM1	S100A8	
BSG	CKLF	GPR15	MAG	S100A9	
C5AR1	COL1A1	GRB14	MERTK	S1PR1	
CALCA	COL1A2	GRB2	MIF	SAA1	
CAV1	CORO1A	GRB7	MITF	SBDS	
CCL1	CSF3R	HCK	MMP1	SCG2	
CCL11	CX3CL1	HIST1H2BA	MMP9	SELE	
CCL13	CX3CR1	HMGB1	MSN	SELL	
CCL14	CXADR	HRAS	MYH9	SELP	
CCL15	CXCL10	HRH1	MYO1G	SELPLG	
CCL16	CXCL11	ICAM1	NCKAP1L	SFTPD	
CCL17	CXCL13	IFNG	NKX2-3	SHC1	
CCL18	CXCL16	IL10	NLRP10	SIRPA	
CCL19	CXCL3	IL16	NLRP12	SIRPG	
CCL2	CXCR1	IL17RA	NRAS	SLC16A1	
CCL20	CXCR2	IL17RC	NUP85	SLC16A3	
CCL21	CXCR3	IL1B	OLR1	SLC16A8	
CCL22	CXCR4	IL6	PDE4B	SLC3A2	
CCL23	DAPK2	IL6R	PDE4D	SLC7A10	
CCL24	DBH	IL8	PDGFB	SLC7A11	
CCL25	DOCK8	INPP5D	PECAM1	SLC7A5	
CCL26	DOK2	IRAK4	PF4	SLC7A6	
CCL3	EDN1	ITGA1	PF4V1	SLC7A7	
CCL3L1	EDN2	ITGA3	PIK3CA	SLC7A8	
CCL3L3	EDN3	ITGA4	PIK3CB	SLC7A9	
CCL4	EDNRB	ITGA5	PIK3CD	SOS1	
CCL4L2	ELANE	ITGA6	PIK3CG	SPN	
CCL5	EMR2	ITGA9	PIK3R1	SPNS2	
CCL7	EPS8	ITGAL	PIK3R2	SRC	
CCL8	EPX	ITGAM	PIP5K1C	STAT5B	
CCR1	ESAM	ITGAV	PLA2G1B	SYK	
CCR2	F11R	ITGAX	PLCG1	TBX21	
CCR5	F2	ITGB1	PODXL	TEK	
CCR6	F2RL1	ITGB2	PODXL2	TGFB2	

Supplementary Table 2. The list of overlapping leukocyte migration related genes associated with tumor purity in both of CGGA and TCGA RNA sequencing datasets.

Negative	Positive
B4GALT1	PODXL2
C5AR1	
CCL5	
CCR1	
CCR2	
CCR7	
CD2	
CD48	
CORO1A	
CXCL16	
DOK2	
FCER1G	
ITGAM	
ITGB2	
LYN	
PECAM1	
PIK3CD	
PROCR	
SLC7A7	
THBD	
LSP1	

Supplementary Table 3. The list of differential expressed leukocyte migration related genes between GBM and LGG, according to CGGA and TCGA RNA sequencing datasets.

AMICA1	GATA3
ANGPT2	GRB14
ANXA1	IL10
CCL13	IL6
CCL18	IL8
CCL20	ITGA4
CCL25	ITGB3
CCL26	LBP
CCL5	LCK
CCL7	LSP1
CCL8	MMP1
CCR2	MMP9
CCR5	MYO1G
CCR7	PPBP
CD244	PROC
CD2	S100A12
CD48	S100A8
CD58	S100A9
CEACAM1	SAA1
COL1A1	SIRPG
CXCL10	SLC7A9
CXCL11	TREM1
CXCL13	VAV3
CXCL3	XCL1
CXCR1	XCL2
CXCR2	ZAP70
CXCR3	EMR2
DOK2	LGALS3
EDN2	THBD
ELANE	GRB7
	SELP

Supplementary Table 4. Univariate Cox regression analyses of leukocyte migration related genes with P < 0.05 in CGGA and TCGA RNA sequencing datasets with the overall survival of GBM patients.

CGGA RNA sequencing dataset			
**Variable**	**HR**	**95%CI**	***P* Value**
CCL5	1.0769	0.9185-1.2625	0.3613
CCR2	1.6333	1.2028-2.2177	0.0017
CCR7	1.3580	1.0034-1.8380	0.0475
CD2	1.0544	0.9848-1.1289	0.1283
CD48	1.1697	1.0402-1.3155	0.0089
DOK2	1.1102	1.0118-1.2183	0.0273
LSP1	1.3475	1.1170-1.6256	0.0018
THBD	1.0150	0.9885-1.0422	0.2695

TCGA RNA sequencing dataset			
**Variable**	**HR**	**95%CI**	***P* Value**
CCL5	1.1307	0.9844	0.0822
CCR2	1.0621	0.9498	0.2908
CCR7	1.1707	1.0140	0.0316
CD2	1.0456	0.9377	0.4222
CD48	1.0372	0.9130	0.5742
DOK2	1.1219	0.9772	0.1024
LSP1	1.3380	1.1176	0.0015
THBD	1.1949	1.0271	0.0211

Supplementary Table 5. The GBM patients' information WHO grade IV (n=53).

Sample NO.	Gender	Age	Chemotherapy	Clinical status	GIS score
G01	F	58	No	Primary	4
G02	F	68	NA	Primary	6
G03	M	46	NA	Primary	3
G04	F	67	NA	Primary	6
G05	F	58	NA	Primary	2
G06	M	53	Yes	Primary	6
G07	F	60	NA	Primary	3
G08	F	69	Yes	Primary	6
G09	M	47	No	Primary	2
G10	F	50	NA	Primary	6
G11	M	71	NA	Primary	9
G12	M	72	No	Primary	6
G13	F	52	No	Primary	9
G14	F	54	NA	Primary	6
G15	M	54	NA	Secondary	2
G16	F	69	NA	Primary	6
G17	M	35	Yes	Primary	3
G18	M	24	NA	Primary	6
G19	M	61	NA	Primary	9
G20	M	63	NA	Primary	6
G21	M	47	Yes	Primary	0
G22	M	38	NA	Primary	0
G23	M	71	Yes	Primary	6
G24	M	57	No	Primary	0
G25	F	60	NA	Primary	4
G26	F	66	NA	Primary	4
G27	M	40	No	Primary	3
G28	M	55	Yes	Primary	6
G29	M	15	NA	Primary	3
G30	M	57	No	Primary	4
G31	F	58	No	Primary	2
G32	F	73	NA	Primary	9
G33	F	62	NA	Primary	6
G34	M	44	No	Primary	3
G35	M	63	No	Primary	4
G36	M	42	NA	Primary	3
G37	F	43	NA	Primary	4
G38	F	66	NA	Secondary	3
G39	M	48	NA	Primary	6
G40	F	44	NA	Primary	2
G41	M	18	NA	Primary	4
G42	M	41	NA	Primary	9
G43	M	54	No	Primary	3
G44	M	49	NA	Primary	4
G45	F	64	NA	Primary	6
G46	M	32	NA	Primary	3
G47	F	57	NA	Primary	3
G48	F	57	No	Primary	3
G49	F	55	No	Primary	2
G50	F	62	NA	Primary	2
G51	M	50	Yes	Primary	3

G52	M	29	Yes	Primary	0
G53	M	64	Yes	Primary	9

Supplementary Table 6. Univariate and multivariate Cox regression analyses of LSP1 expression with the overall survival of GBM patients (CGGA RNA sequencing, TCGA RNA sequencing, TCGA 4502A and U133 mRNA microarray datasets).

CGGA RNA sequencing database						
**Variables**	**Univariate analysis**			**Multivariate analysis**		
	**HR**	**95%CI**	**p Value**	**HR**	**95%CI**	***P* Value**
Radiotherapy	0.4119	0.2594-0.6539	0.0002	0.4200	0.2582-0.6832	0.0005
Chemotherapy	0.3359	0.2141-0.5271	0.0000	0.4080	0.2554-0.6518	0.0000
LSP1	1.3475	1.1170-1.6256	0.0018	1.3174	1.0416-1.6663	0.0214

TCGA RNA sequencing database						
**Variables**	**Univariate analysis**			**Multivariate analysis**		
	**HR**	**95%CI**	**p Value**	**HR**	**95%CI**	***P* Value**
Radiotherapy	0.1629	0.0984-0.2697	0.0000	0.2209	0.0919-0.5313	0.0007
Chemotherapy	0.2629	0.1662-0.4159	0.0000	0.7190	0.3287-1.5727	0.4088
LSP1	1.3380	1.1176-1.6017	0.0015	1.3124	1.0697-1.6102	0.0092

TCGA mRNA 4502A database						
**Variables**	**Univariate analysis**			**Multivariate analysis**		
	**HR**	**95%CI**	**p Value**	**HR**	**95%CI**	***P* Value**
Radiotherapy	0.1281	0.0946-0.1734	0.0000	0.1645	0.1142-0.2370	0.0000
Chemotherapy	0.4166	0.3220-0.5390	0.0000	0.6891	0.5074-0.9358	0.0171
LSP1	1.4232	1.2065-1.6787	0.0000	1.4616	1.2224-1.7476	0.0000

TCGA mRNA U133 database						
**Variables**	**Univariate analysis**			**Multivariate analysis**		
	**HR**	**95%CI**	**p Value**	**HR**	**95%CI**	***P* Value**
Radiotherapy	0.1295	0.0975-0.1720	0.0000	6.6374	4.6804-9.4127	0.0000
Chemotherapy	0.3950	0.3091-0.5048	0.0000	0.6826	0.5089-0.9155	0.0108
LSP1	1.2276	1.0793-1.3963	0.0018	1.2136	1.0493-1.4036	0.0091

Please browse Full Text version to see the data of Supplementary Tables 7, 8

Supplementary Table 7. The list of genes positively correlated with *LSP1* expression both in CGGA and TCGA RNA sequencing datasets.

Supplementary Table 8. The list of 892 overlapping upregulated genes correlated to high LSP1 expression in CGGA and TCGA RNA sequencing datasets.

Supplementary Table 9. The list of immunosuppressive genes.

PD-L1
CD276
CD28
CD80
CD86
CTLA4
HAVCR2
HHLA2
ICOSLG
ICOS
LAG3
LAIR1
LAIR2
LILRA1
LILRA2
LILRA3
LILRA4
LILRA5
LILRA6
LILRB1
LILRB2
LILRB3
LILRB4
LILRB5
LILRP2
OSMR
OSM
PD-L2
PDCD1
PDCD6
TMIGD2
VTCN1
ADORA2A
TIGIT
TNFRSF18
TNFRSF4
TNFRSF9
AIMP1
CCL18
FCGR2B
FOXO3
IL10
IL6
MMP9
ZBTB16
