# Supplementary material for: Elevated lymphocyte specific protein 1 expression is involved in the regulation of leukocyte migration and immunosuppressive microenvironment in glioblastoma
Source: Aging (Albany NY). 2020 Jan 29;12(2):1656–84. doi: 10.18632/aging.102706 (PMC7053627; doi:10.18632/aging.102706)
Supplement: Supplementary Table 7 [file aging-12-102706-s001..docx]

| **Supplementary Table 7. The list of genes positively correlated with LSP1 expression both in CGGA and TCGA RNA sequencing datasets.** | | | | | | | | |
| --- | --- | --- | --- | --- | --- | --- | --- | --- |
| **TCGA** | | | | |  | **CGGA** | | |
| ABI3 |  | GNA15 |  | RNASE2 |  |  | ADAP2 |  |
| ABTB1 |  | GNGT2 |  | RNASE3 |  |  | ADPRH |  |
| ACAP1 |  | GPNMB |  | RNASE6 |  |  | APOBEC3C |  |
| ACP5 |  | GPR132 |  | RNASET2 |  |  | APOBR |  |
| ACPP |  | GPR183 |  | RNF135 |  |  | AQP9 |  |
| ACSL1 |  | GPR65 |  | RNF144B |  |  | ARHGAP30 |  |
| ADAM8 |  | GPR77 |  | RPS6KA1 |  |  | ARID3A |  |
| ADAP2 |  | GPR84 |  | RUNX2 |  |  | ARL11 |  |
| ADORA3 |  | GPRIN3 |  | S100A11 |  |  | ARPC1B |  |
| AIF1 |  | GPSM3 |  | S100A4 |  |  | ATF5 |  |
| ALDH3B1 |  | GRN |  | S100A8 |  |  | B4GALT1 |  |
| ALOX5AP |  | HAMP |  | S100A9 |  |  | BACE2 |  |
| ALOX5 |  | HAVCR2 |  | SAMSN1 |  |  | BCL2A1 |  |
| AMPD3 |  | HCK |  | SASH3 |  |  | BCL3 |  |
| ANXA11 |  | HCLS1 |  | SAT1 |  |  | C15orf48 |  |
| ANXA2P1 |  | HCST |  | SCIN |  |  | CAPZA1 |  |
| ANXA2P2 |  | HK3 |  | SERPINA1 |  |  | CASP4 |  |
| ANXA2 |  | HLA.DMA |  | SERPINB1 |  |  | CCDC109B |  |
| APOB48R |  | HLA.DMB |  | SH2D2A |  |  | CCRL2 |  |
| AQP9 |  | HMHA1 |  | SH3BGRL3 |  |  | CD300A |  |
| ARHGAP30 | | HMOX1 |  | SH3BP1 |  |  | CD300C |  |
| ARHGAP4 |  | ICAM1 |  | SH3TC1 |  |  | CD300LF |  |
| ARHGAP9 |  | ICOSLG |  | SIGLEC7 |  |  | CD40 |  |
| ARHGDIB |  | IER3 |  | SIGLEC9 |  |  | CD44 |  |
| ARL11 |  | IFI30 |  | SIGLECP3 |  |  | CD68 |  |
| ARPC1B |  | IL10RA |  | SIPA1 |  |  | CDCP1 |  |
| ARRB2 |  | IL10 |  | SIRPB2 |  |  | CHPF2 |  |
| ASCL2 |  | IL16 |  | SLAMF8 |  |  | CLDN23 |  |
| ASGR2 |  | IL1R1 |  | SLA |  |  | CLIC1 |  |
| B3GNT8 |  | IL1R2 |  | SLC11A1 |  |  | CSF2RB |  |
| B4GALT1 |  | IL1RN |  | SLC15A3 |  |  | CSTA |  |
| BATF |  | IL2RG |  | SLC16A3 |  |  | CTSB |  |
| BCL2A1 |  | IL4I1 |  | SLC17A9 |  |  | CTSC |  |
| BCL3 |  | IL4R |  | SLC2A5 |  |  | CTSD |  |
| BTK |  | IRF5 |  | SLC31A2 |  |  | CTSL1 |  |
| C15orf48 |  | ITGA5 |  | SLC37A2 |  |  | CTSZ |  |
| C16orf54 |  | ITGAM |  | SLC6A6 |  |  | CYP2S1 |  |
| C17orf60 |  | ITGB2 |  | SLC7A7 |  |  | CYTH4 |  |
| C17orf87 |  | KCNK13 |  | SNAI3 |  |  | CYTIP |  |
| C19orf59 |  | KCNK6 |  | SOCS3 |  |  | DNAJC5B |  |
| C1QA |  | KCNQ1 |  | SPI1 |  |  | DOCK2 |  |
| C1QB |  | KLHL6 |  | SPINT1 |  |  | DOK3 |  |
| C1QC |  | KMO |  | SPINT2 |  |  | EDEM1 |  |
| C1S |  | KYNU |  | SPN |  |  | EHBP1L1 |  |
| C1orf162 |  | LACTB |  | SPP1 |  |  | EMR3 |  |
| C1orf38 |  | LAIR1 |  | SQRDL |  |  | FBP1 |  |
| C2 |  | LAPTM5 |  | SRGN |  |  | FCER1G |  |
| C3AR1 |  | LCP1 |  | ST14 |  |  | FERMT3 |  |
| C5AR1 |  | LCP2 |  | ST8SIA4 |  |  | FGR |  |
| C6orf105 |  | LGALS9 |  | STAB1 |  |  | FPR1 |  |
| C9orf167 |  | LHFPL2 |  | STAC3 |  |  | FPR2 |  |
| CACNA2D4 | | LILRA2 |  | STARD5 |  |  | FTL |  |
| CAPG |  | LILRA3 |  | STAT6 |  |  | FUCA2 |  |
| CATSPER1 |  | LILRA5 |  | STX11 |  |  | GLIPR1 |  |
| CCDC69 |  | LILRA6 |  | STXBP2 |  |  | GLT25D1 |  |
| CCL2 |  | LILRB1 |  | SYK |  |  | GRN |  |
| CCR1 |  | LILRB2 |  | SYNGR2 |  |  | HAVCR2 |  |
| CCR5 |  | LILRB3 |  | SYTL3 |  |  | HEXB |  |
| CCRL2 |  | LILRB4 |  | TACSTD2 |  |  | HK3 |  |
| CD14 |  | LITAF |  | TBC1D10C |  |  | HSD3B7 |  |
| CD163 |  | LOC100233209 | | TBC1D22A |  |  | IFI30 |  |
| CD180 |  | LOC606724 |  | TBXAS1 |  |  | IL17RA |  |
| CD1D |  | LSP1 |  | TCIRG1 |  |  | IL4R |  |
| CD28 |  | LPXN |  | TEC |  |  | IQGAP1 |  |
| CD300A |  | LRG1 |  | TGFB1 |  |  | ITGA5 |  |
| CD300C |  | LRRC25 |  | TGFBI |  |  | ITGB2 |  |
| CD300E |  | LRRC33 |  | THBD |  |  | KCNK6 |  |
| CD300LB |  | LTBP2 |  | TIMP1 |  |  | LAPTM5 |  |
| CD300LF |  | LTBR |  | TLR1 |  |  | LCP1 |  |
| CD33 |  | LYZ |  | TLR2 |  |  | LCP2 |  |
| CD37 |  | MAFB |  | TLR5 |  |  | LHFPL2 |  |
| CD40 |  | MAN1A1 |  | TLR8 |  |  | LILRB3 |  |
| CD4 |  | MANBA |  | TM7SF4 |  |  | LOC100506585 | |
| CD52 |  | MAP3K8 |  | TMC8 |  |  | LSP1 |  |
| CD53 |  | MAPK13 |  | TMEM106A | |  | LY96 |  |
| CD55 |  | MEI1 |  | TMEM150B | |  | MAN2B1 |  |
| CD68 |  | METRNL |  | TNFAIP8 |  |  | MANBA |  |
| CD72 |  | MFSD1 |  | TNFRSF11A | |  | MFSD7 |  |
| CD7 |  | MFSD7 |  | TNFRSF1B |  |  | MGAT1 |  |
| CD86 |  | MGAT1 |  | TNFSF12.TNFSF13 | | | MLKL |  |
| CDCP1 |  | MLPH |  | TNFSF12 |  |  | MSN |  |
| CEACAM4 |  | MMP7 |  | TNFSF13 |  |  | MSR1 |  |
| CEBPB |  | MPP1 |  | TNFSF8 |  |  | MYH9 |  |
| CEBPD |  | MS4A4A |  | TNFSF9 |  |  | MYL12A |  |
| CFD |  | MS4A6A |  | TNNI2 |  |  | MYO1E |  |
| CFI |  | MS4A7 |  | TRAF3IP3 |  |  | MYO1F |  |
| CLDN23 |  | MSR1 |  | TREM1 |  |  | MYO1G |  |
| CLEC5A |  | MYO1F |  | TREML2 |  |  | NCF1 |  |
| CLEC7A |  | MYO1G |  | TREML3 |  |  | NPC2 |  |
| CMKLR1 |  | NAGA |  | TRPM2 |  |  | OSTF1 |  |
| CMTM7 |  | NCF1C |  | TRPV2 |  |  | PARVG |  |
| CORO1A |  | NCF1 |  | TYMP |  |  | PLA2G15 |  |
| CPPED1 |  | NCF2 |  | TYROBP |  |  | PLAUR |  |
| CR1 |  | NCF4 |  | VAMP8 |  |  | PLEK |  |
| CREG1 |  | NCKAP1L |  | VASP |  |  | PLIN2 |  |
| CSF1R |  | NDRG1 |  | VAV1 |  |  | PLK3 |  |
| CSF2RB |  | NFAM1 |  | VDR |  |  | PTRF |  |
| CSF3R |  | NINJ1 |  | VMO1 |  |  | RAB42 |  |
| CST7 |  | NLRC4 |  | VSIG4 |  |  | RAC2 |  |
| CSTA |  | NLRP3 |  | WAS |  |  | RASSF3 |  |
| CTSB |  | NOD2 |  | WIPF1 |  |  | RBM47 |  |
| CTSC |  | NPC2 |  | WIPI1 |  |  | RELB |  |
| CTSD |  | NPL |  |  |  |  | RIPK3 |  |
| CTSL1 |  | NTAN1 |  |  |  |  | RNF149 |  |
| CTSS |  | NUDT16P1 |  |  |  |  | S100A11 |  |
| CTSZ |  | OSCAR |  |  |  |  | S100A9 |  |
| CXCL16 |  | OSM |  |  |  |  | SASH3 |  |
| CYBA |  | OSTF1 |  |  |  |  | SAT1 |  |
| CYP27A1 |  | P2RX4 |  |  |  |  | SERPINE1 |  |
| CYP2S1 |  | P4HA2 |  |  |  |  | SH2B3 |  |
| CYTH4 |  | PARVG |  |  |  |  | SIGLEC5 |  |
| CYTIP |  | PDE6G |  |  |  |  | SIGLEC7 |  |
| DAB2 |  | PDK3 |  |  |  |  | SIPA1 |  |
| DEF6 |  | PIK3AP1 |  |  |  |  | SLC11A1 |  |
| DENND1C |  | PIK3R5 |  |  |  |  | SLC15A3 |  |
| DENND2D |  | PILRA |  |  |  |  | SPI1 |  |
| DENND3 |  | PLAUR |  |  |  |  | SPP1 |  |
| DOCK2 |  | PLB1 |  |  |  |  | SQRDL |  |
| DOK1 |  | PLBD1 |  |  |  |  | ST14 |  |
| DOK2 |  | PLCG2 |  |  |  |  | ST8SIA4 |  |
| DOK3 |  | PLEKHO2 |  |  |  |  | STAT5A |  |
| ELF4 |  | PLEK |  |  |  |  | STX11 |  |
| EMB |  | PLIN2 |  |  |  |  | TCIRG1 |  |
| EMR1 |  | PLK3 |  |  |  |  | TGFB1 |  |
| EMR2 |  | PLTP |  |  |  |  | THEMIS2 |  |
| EVI2B |  | PLXDC2 |  |  |  |  | TLR2 |  |
| FAH |  | PPARG |  |  |  |  | TMBIM1 |  |
| FAM113B |  | PRAM1 |  |  |  |  | TNFRSF10A |  |
| FAM20A |  | PRKCD |  |  |  |  | TNFRSF12A |  |
| FBP1 |  | PTGER2 |  |  |  |  | TNFRSF14 |  |
| FCAR |  | PTPN18 |  |  |  |  | TREML2 |  |
| FCER1G |  | PTPN22 |  |  |  |  | TRIM38 |  |
| FCGBP |  | PTPN6 |  |  |  |  | TRPV2 |  |
| FCGR2A |  | PTPN7 |  |  |  |  | TUBA1C |  |
| FCGR2B |  | PTPRC |  |  |  |  | TYROBP |  |
| FCGR2C |  | PYCARD |  |  |  |  | VAMP8 |  |
| FCGR3A |  | RAB11FIP1 | |  |  |  | ZNF600 |  |
| FERMT3 |  | RAB27A |  |  |  |  |  |  |
| FES |  | RAB42 |  |  |  |  |  |  |
| FGR |  | RAC2 |  |  |  |  |  |  |
| FMNL1 |  | RAP2B |  |  |  |  |  |  |
| FPR1 |  | RARRES1 |  |  |  |  |  |  |
| FPR2 |  | RASGRP4 |  |  |  |  |  |  |
| FPR3 |  | RASSF5 |  |  |  |  |  |  |
| FTL |  | RBM47 |  |  |  |  |  |  |
| FUCA1 |  | RGS19 |  |  |  |  |  |  |
| FXYD5 |  | RHOG |  |  |  |  |  |  |
| GALNT6 |  | RIN3 |  |  |  |  |  |  |
| GAS6 |  | RIPK3 |  |  |  |  |  |  |
| GBGT1 |  | RNASE1 |  |  |  |  |  |  |
