# Supplementary material for: Elevated lymphocyte specific protein 1 expression is involved in the regulation of leukocyte migration and immunosuppressive microenvironment in glioblastoma
Source: Aging (Albany NY). 2020 Jan 29;12(2):1656–84. doi: 10.18632/aging.102706 (PMC7053627; doi:10.18632/aging.102706)
Supplement: Supplementary Table 8 [file aging-12-102706-s002..docx]

| **Supplementary Table 8. The list of 892 overlapping upregulated genes correlated to high LSP1 expression in CGGA and TCGA RNA sequencing datasets.** | | | | | | | | |
| --- | --- | --- | --- | --- | --- | --- | --- | --- |
|  |  |  |  |  |  |  |  |  |
| A2M |  | CSTB |  | IL8 |  | PMFBP1 |  | TGFB1 |
| ABCC3 |  | CTBS |  | IMPA2 |  | PMP22 |  | TGFBI |
| ABCD1 |  | CTSA |  | IMPDH1 |  | PODNL1 |  | TGFBR2 |
| ABHD14B |  | CTSB |  | INPP5D |  | POLD4 |  | TGM2 |
| ABHD15 |  | CTSC |  | IRAK3 |  | PPCS |  | THBD |
| ABI3 |  | CTSD |  | IRF1 |  | PPM1M |  | THBS1 |
| ACAP1 |  | CTSL1 |  | IRF5 |  | PPP1R15A |  | TIFA |
| ACP2 |  | CTSS |  | IRF8 |  | PQLC3 |  | TIMP1 |
| ACP5 |  | CTSZ |  | ISG20 |  | PRAM1 |  | TLR1 |
| ACPP |  | CXCL14 |  | ITGA4 |  | PRDM1 |  | TLR2 |
| ACSL5 |  | CXCL16 |  | ITGA5 |  | PRF1 |  | TLR5 |
| ACTA2 |  | CXCL1 |  | ITGAL |  | PRICKLE3 |  | TLR6 |
| ACTB |  | CXCL2 |  | ITGAM |  | PRKCD |  | TLR7 |
| ADAM12 |  | CXCL6 |  | ITGAX |  | PROCR |  | TLR8 |
| ADAM28 |  | CXCR1 |  | ITGB1 |  | PROS1 |  | TM6SF1 |
| ADAM8 |  | CXCR4 |  | ITGB2 |  | PRPS2 |  | TMBIM1 |
| ADAMTS1 |  | CXorf21 |  | ITGB3 |  | PRR13 |  | TMC8 |
| ADAP2 |  | CYBA |  | ITGB5 |  | PRSS23 |  | TMED9 |
| ADCK2 |  | CYBB |  | ITGB7 |  | PSD4 |  | TMEM106A |
| ADORA3 |  | CYP1B1 |  | ITPKC |  | PSMB10 |  | TMEM109 |
| ADPGK |  | CYP27A1 |  | JAK3 |  | PTAFR |  | TMEM140 |
| ADPRH |  | CYP2S1 |  | JUNB |  | PTGER2 |  | TMEM150A |
| AGPAT2 |  | CYR61 |  | KCNE1 |  | PTGER4 |  | TMEM173 |
| AHNAK2 |  | CYTH4 |  | KCNE3 |  | PTGES |  | TMEM176A |
| AIF1 |  | CYTIP |  | KCNJ5 |  | PTGIR |  | TMEM176B |
| AIFM2 |  | DAB2 |  | KCNK13 |  | PTPLAD2 |  | TMEM37 |
| AIM1 |  | DAPP1 |  | KCNK6 |  | PTPN22 |  | TMEM70 |
| ALOX15B |  | DDB2 |  | KCNQ1 |  | PTPN6 |  | TMEM86A |
| ALOX5AP |  | DEF6 |  | KCTD10 |  | PTPN7 |  | TNFAIP2 |
| ALPK1 |  | DENND1C |  | KIAA0247 |  | PTPRC |  | TNFAIP3 |
| AMICA1 |  | DENND2D |  | KLHL6 |  | PTPRH |  | TNFAIP8L3 |
| AMPD3 |  | DENND3 |  | KYNU |  | PTRF |  | TNFAIP8 |
| ANGPTL4 |  | DIRAS3 |  | LACTB |  | PTX3 |  | TNFRSF10A |
| ANPEP |  | DNAJB1 |  | LAIR1 |  | PVRL2 |  | TNFRSF10B |
| ANXA1 |  | DNAJC21 |  | LAPTM5 |  | PYCARD |  | TNFRSF10C |
| ANXA2 |  | DNAJC5B |  | LAT2 |  | PYGL |  | TNFRSF10D |
| ANXA4 |  | DNASE1L1 |  | LAYN |  | RAB11FIP1 | | TNFRSF14 |
| APBB1IP |  | DNASE2 |  | LCK |  | RAB20 |  | TNFRSF1A |
| APEX2 |  | DOCK2 |  | LCP1 |  | RAB27A |  | TNFRSF1B |
| APOBEC3C | | DOK1 |  | LCP2 |  | RAB38 |  | TNFSF12 |
| APOBEC3F | | DOK2 |  | LENG9 |  | RAB42 |  | TNFSF13 |
| APOBEC3G | | DOK3 |  | LEPREL1 |  | RAC2 |  | TNFSF8 |
| APOL3 |  | DPEP2 |  | LGALS1 |  | RAP2B |  | TNIP1 |
| AQP9 |  | DPP4 |  | LGALS9 |  | RASAL3 |  | TPK1 |
| ARAP1 |  | DPYD |  | LHFPL2 |  | RASSF3 |  | TPP1 |
| ARHGAP15 | | DRAM1 |  | LIF |  | RASSF5 |  | TPRG1 |
| ARHGAP18 | | DSC2 |  | LILRA2 |  | RBM47 |  | TRADD |
| ARHGAP29 | | DSE |  | LILRA6 |  | RBPMS |  | TRAF3IP3 |
| ARHGAP30 | | DTX2 |  | LILRB1 |  | RCAN3 |  | TRAT1 |
| ARHGAP9 |  | DUSP1 |  | LILRB3 |  | RCSD1 |  | TREM1 |
| ARHGDIB |  | DUSP3 |  | LIMK2 |  | RDH10 |  | TREM2 |
| ARHGEF5 |  | DYNLT3 |  | LIPN |  | REEP4 |  | TREML2 |
| ARID3A |  | EFEMP2 |  | LITAF |  | RELB |  | TRIM38 |
| ARID5A |  | EHBP1L1 |  | LOXL1 |  | REL |  | TRIP4 |
| ARL11 |  | EHD2 |  | LOXL2 |  | RENBP |  | TRPV2 |
| ARPC1B |  | EHD4 |  | LOXL3 |  | RETN |  | TRPV4 |
| ARRB2 |  | ELF4 |  | LOX |  | RFTN1 |  | TSLP |
| ARRDC2 |  | ELK3 |  | LPAR5 |  | RGS16 |  | TSPAN4 |
| ASL |  | ELL2 |  | LPXN |  | RGS18 |  | TSPO |
| ATF5 |  | EMB |  | LRG1 |  | RGS19 |  | TTC38 |
| ATG7 |  | EMILIN2 |  | LRRC25 |  | RGS1 |  | TTC7A |
| ATP2A3 |  | EMP3 |  | LRRC32 |  | RHBDF2 |  | TUBA1C |
| ATP6V0D2 |  | EMR1 |  | LRRC33 |  | RHOG |  | TWF2 |
| ATP8B1 |  | EMR2 |  | LST1 |  | RILPL2 |  | TXK |
| ATP8B3 |  | EMR3 |  | LTBR |  | RILP |  | TXNDC11 |
| AXL |  | EPHX3 |  | LTB |  | RIN3 |  | TYMP |
| B3GALT4 |  | ERO1L |  | LY75 |  | RIPK3 |  | TYROBP |
| B3GNT8 |  | ESYT1 |  | LY86 |  | RNASE2 |  | UCP2 |
| B4GALT1 |  | EVI2B |  | LY96 |  | RNASE3 |  | UNC93B1 |
| BACE2 |  | F11R |  | LYL1 |  | RNASE4 |  | UPP1 |
| BATF |  | F13A1 |  | LYN |  | RNASE6 |  | VAMP5 |
| BCL2A1 |  | FAH |  | LYPD3 |  | RNASET2 |  | VAMP8 |
| BCL3 |  | FAM109B |  | LYZ |  | RNF135 |  | VASP |
| BDKRB2 |  | FAM114A1 |  | MAFB |  | RNF144B |  | VAV1 |
| BET1L |  | FAM129B |  | MAFF |  | RNF149 |  | VDR |
| BHLHE40 |  | FAM159A |  | MAFK |  | RNF19B |  | VIM |
| BHLHE41 |  | FAM20A |  | MALT1 |  | RNLS |  | VMO1 |
| BIN2 |  | FAM20C |  | MAN1A1 |  | RNPEPL1 |  | VNN1 |
| BIRC3 |  | FAM26F |  | MAN1C1 |  | RPGRIP1 |  | VNN2 |
| BLVRB |  | FAM3B |  | MAN2A1 |  | RPL39L |  | VNN3 |
| BMP2K |  | FAM78A |  | MAN2B1 |  | RPS6KA1 |  | VSIG4 |
| BRI3 |  | FAM83G |  | MANBA |  | RRAS |  | VWA1 |
| BTK |  | FASLG |  | MAP2K3 |  | RUNX1 |  | WAS |
| C10orf10 |  | FBP1 |  | MAP3K3 |  | RUNX2 |  | WDFY4 |
| C10orf11 |  | FCER1G |  | MAP3K6 |  | S100A10 |  | WDR1 |
| C11orf75 |  | FCGBP |  | MAP3K8 |  | S100A11 |  | WIPI1 |
| C15orf48 |  | FCGR1A |  | MAPK13 |  | S100A4 |  | XKR8 |
| C16orf54 |  | FCGR1C |  | MAPKAPK2 | | S100A8 |  | YIPF1 |
| C19orf38 |  | FCGR2A |  | MBD2 |  | S100A9 |  | ZBTB7B |
| C19orf59 |  | FCGR3B |  | MEFV |  | S1PR4 |  | ZC3H12A |
| C1QA |  | FCGRT |  | MEI1 |  | SAMSN1 |  | ZDHHC12 |
| C1QB |  | FCN1 |  | MESDC1 |  | SASH3 |  | ZNRF2 |
| C1QC |  | FERMT3 |  | METRNL |  | SAT1 |  |  |
| C1RL |  | FGL2 |  | MFSD1 |  | SCIN |  |  |
| C1R |  | FGR |  | MFSD7 |  | SCPEP1 |  |  |
| C1S |  | FHL2 |  | MGAT1 |  | SDC2 |  |  |
| C1orf162 |  | FHOD1 |  | MGAT4B |  | SDS |  |  |
| C1orf85 |  | FKBP15 |  | MICALL2 |  | SEC24D |  |  |
| C2 |  | FKBP9 |  | MLKL |  | SECTM1 |  |  |
| C3AR1 |  | FMNL1 |  | MMP10 |  | SEL1L3 |  |  |
| C3 |  | FN1 |  | MMP11 |  | SEPHS2 |  |  |
| C5AR1 |  | FOSL1 |  | MMP14 |  | SERPINA1 |  |  |
| C9orf64 |  | FOSL2 |  | MMP19 |  | SERPINB1 |  |  |
| CA12 |  | FPR1 |  | MMP7 |  | SERPINB6 |  |  |
| CALHM2 |  | FPR2 |  | MMP8 |  | SERPINB9 |  |  |
| CAPG |  | FPR3 |  | MNDA |  | SERPINE1 |  |  |
| CAPZA1 |  | FRRS1 |  | MPEG1 |  | SERPING1 |  |  |
| CARD11 |  | FTH1 |  | MR1 |  | SERTAD3 |  |  |
| CARD6 |  | FTL |  | MS4A4A |  | SFT2D2 |  |  |
| CASP4 |  | FUCA1 |  | MS4A6A |  | SGMS2 |  |  |
| CASP8 |  | FURIN |  | MSN |  | SH2B3 |  |  |
| CAST |  | FXYD5 |  | MSR1 |  | SH2D2A |  |  |
| CATSPER1 |  | G0S2 |  | MVP |  | SH2D4A |  |  |
| CBLN3 |  | GAA |  | MXRA5 |  | SH3BGRL3 |  |  |
| CCDC109B |  | GAL3ST4 |  | MYADM |  | SH3TC1 |  |  |
| CCDC69 |  | GALC |  | MYH9 |  | SHKBP1 |  |  |
| CCL20 |  | GALM |  | MYL12A |  | SIGIRR |  |  |
| CCL23 |  | GALNT12 |  | MYL9 |  | SIGLEC10 |  |  |
| CCL26 |  | GALNT2 |  | MYO1C |  | SIGLEC15 |  |  |
| CCL2 |  | GALNT5 |  | MYO1E |  | SIGLEC5 |  |  |
| CCL5 |  | GALNT6 |  | MYO1F |  | SIGLEC7 |  |  |
| CCL7 |  | GAPT |  | MYO1G |  | SIGLEC9 |  |  |
| CCR1 |  | GAS6 |  | MYOF |  | SIL1 |  |  |
| CCR2 |  | GBP2 |  | NAGA |  | SIPA1 |  |  |
| CCR7 |  | GCLM |  | NAIP |  | SIRPB2 |  |  |
| CCRL2 |  | GCNT1 |  | NAMPT |  | SIT1 |  |  |
| CCRN4L |  | GGA2 |  | NANS |  | SLAMF8 |  |  |
| CD163 |  | GGN |  | NCF1B |  | SLAMF9 |  |  |
| CD180 |  | GIMAP4 |  | NCF1C |  | SLA |  |  |
| CD1D |  | GIMAP6 |  | NCF1 |  | SLC10A3 |  |  |
| CD226 |  | GLA |  | NCF2 |  | SLC11A1 |  |  |
| CD248 |  | GLIPR1 |  | NCF4 |  | SLC12A7 |  |  |
| CD2 |  | GLT25D1 |  | NCKAP1L |  | SLC15A3 |  |  |
| CD300A |  | GM2A |  | NDRG1 |  | SLC16A10 |  |  |
| CD300C |  | GMFG |  | NFAM1 |  | SLC16A13 |  |  |
| CD300E |  | GNA15 |  | NFE2L3 |  | SLC16A3 |  |  |
| CD300LB |  | GPNMB |  | NFKB1 |  | SLC22A18 |  |  |
| CD300LF |  | GPR141 |  | NFKBIZ |  | SLC24A6 |  |  |
| CD33 |  | GPR157 |  | NKG7 |  | SLC25A19 |  |  |
| CD37 |  | GPR160 |  | NLRC4 |  | SLC25A24 |  |  |
| CD3E |  | GPR171 |  | NNMT |  | SLC25A43 |  |  |
| CD3G |  | GPR65 |  | NOD1 |  | SLC29A1 |  |  |
| CD40 |  | GPR84 |  | NOD2 |  | SLC2A3 |  |  |
| CD44 |  | GPRC5A |  | NPC2 |  | SLC2A5 |  |  |
| CD48 |  | GPRIN3 |  | NRP1 |  | SLC2A9 |  |  |
| CD4 |  | GPX1 |  | NTAN1 |  | SLC35C1 |  |  |
| CD52 |  | GPX8 |  | NUAK2 |  | SLC37A2 |  |  |
| CD55 |  | GRAP2 |  | NUCB1 |  | SLC39A8 |  |  |
| CD59 |  | GRN |  | NUDT16P1 |  | SLC43A3 |  |  |
| CD5 |  | GTSF1 |  | NXF3 |  | SLC46A2 |  |  |
| CD68 |  | GUSB |  | OLR1 |  | SLC46A3 |  |  |
| CD69 |  | GXYLT2 |  | OPLAH |  | SLC4A3 |  |  |
| CD6 |  | GYPC |  | OSMR |  | SLC7A7 |  |  |
| CD72 |  | H2AFJ |  | OSM |  | SLC9A1 |  |  |
| CD84 |  | HAAO |  | OSTF1 |  | SMPDL3A |  |  |
| CD86 |  | HAVCR2 |  | P2RY10 |  | SNAI1 |  |  |
| CD93 |  | HCLS1 |  | P2RY8 |  | SNX20 |  |  |
| CD96 |  | HCST |  | P4HA1 |  | SNX9 |  |  |
| CDCP1 |  | HDAC7 |  | P4HA2 |  | SOAT1 |  |  |
| CDKN1A |  | HDHD3 |  | P4HB |  | SOCS3 |  |  |
| CEACAM21 | | HEXA |  | PARP15 |  | SP100 |  |  |
| CEACAM4 |  | HEXB |  | PARVB |  | SP140 |  |  |
| CEBPD |  | HGSNAT |  | PARVG |  | SPAG4 |  |  |
| CFD |  | HHEX |  | PAX8 |  | SPATS2L |  |  |
| CFI |  | HK3 |  | PCK2 |  | SPI1 |  |  |
| CFLAR |  | HLX |  | PDCD1LG2 | | SPINK1 |  |  |
| CHI3L1 |  | HMHA1 |  | PDCD1 |  | SPINT1 |  |  |
| CHST2 |  | HMOX1 |  | PDE6G |  | SPN |  |  |
| CHSY1 |  | HPGDS |  | PDIA3 |  | SPOCD1 |  |  |
| CIDEB |  | HPS1 |  | PECAM1 |  | SPP1 |  |  |
| CLCF1 |  | HPS3 |  | PELO |  | SQRDL |  |  |
| CLDN23 |  | HPSE |  | PFKL |  | SQSTM1 |  |  |
| CLEC4A |  | HSD3B7 |  | PFN1 |  | SRGN |  |  |
| CLEC5A |  | HSPA5 |  | PGK1 |  | SRPX2 |  |  |
| CLEC7A |  | HSPA6 |  | PIGB |  | SSH3 |  |  |
| CLIC1 |  | HSPB1 |  | PIK3AP1 |  | ST14 |  |  |
| CLN6 |  | HTRA4 |  | PIK3CD |  | ST8SIA4 |  |  |
| CMTM7 |  | ICAM1 |  | PIK3R6 |  | STAB1 |  |  |
| CNN2 |  | ICAM3 |  | PILRA |  | STARD5 |  |  |
| COL18A1 |  | IFI30 |  | PIM3 |  | STAT5A |  |  |
| COL6A1 |  | IFITM2 |  | PLA2G15 |  | STBD1 |  |  |
| COL6A2 |  | IFNGR2 |  | PLA2R1 |  | STC1 |  |  |
| COL8A1 |  | IGFBP6 |  | PLAUR |  | STEAP3 |  |  |
| CORO1A |  | IGSF6 |  | PLAU |  | STK10 |  |  |
| COTL1 |  | IKBIP |  | PLBD1 |  | STX11 |  |  |
| CPD |  | IL10RA |  | PLBD2 |  | SUSD2 |  |  |
| CPM |  | IL10RB |  | PLCG2 |  | SWAP70 |  |  |
| CPPED1 |  | IL13RA1 |  | PLD3 |  | SYNGR2 |  |  |
| CPVL |  | IL15RA |  | PLEK2 |  | SYNPO |  |  |
| CREB3L2 |  | IL18 |  | PLEKHO2 |  | TAGAP |  |  |
| CREG1 |  | IL1A |  | PLEK |  | TAGLN2 |  |  |
| CRTAM |  | IL1B |  | PLIN2 |  | TAGLN |  |  |
| CRTAP |  | IL1R1 |  | PLK3 |  | TBC1D10C |  |  |
| CRYBB1 |  | IL1RN |  | PLOD1 |  | TBC1D1 |  |  |
| CSF1R |  | IL2RA |  | PLOD2 |  | TCIRG1 |  |  |
| CSF2RA |  | IL2RB |  | PLOD3 |  | TCN1 |  |  |
| CSF2RB |  | IL2RG |  | PLP2 |  | TEC |  |  |
| CSF3R |  | IL4I1 |  | PLXDC2 |  | TES |  |  |
| CST7 |  | IL4R |  | PLXNB2 |  | TFAP2C |  |  |
| CSTA |  | IL7R |  | PLXND1 |  | TFEC |  |  |
